# Model‐Based Optimization of Fed‐Batch In Vitro Transcription

**DOI:** 10.1002/cbic.202500485

**Published:** 2025-10-09

**Authors:** Nathan Merica Stover, Soroush Ahmadi, Jacob Rosenfeld, Francesco Destro, Allan S. Myerson, Richard D. Braatz

**Affiliations:** ^1^ Department of Chemical Engineering Massachusetts Institute of Technology 77 Massachusetts Avenue Cambridge MA 02139 USA

**Keywords:** co‐transcriptional capping, in vitro transcription, magnesium phosphate, mRNA synthesis, optimal control

## Abstract

Recent developments in RNA vaccines and therapeutics have motivated the need for process engineering strategies to optimize the in vitro transcription (IVT) reaction for RNA synthesis. Specifically, practitioners seek to maximize the production of RNA and the incorporation of the 5‐prime cap to the end of each RNA molecule while minimizing the use of expensive reagents. Fed‐batch IVT is a promising technique for achieving these goals but is difficult to optimize by purely experimental means. Herein, a mechanistic model for fed‐batch IVT is developed and it is used to develop optimized fed‐batch protocols to maximize the formation of RNA while controlling concentrations of nucleoside triphosphates. On a model sequence that has been shown to be sensitive to salt concentrations, this approach can produce twice as much RNA as a heuristic approach. In addition, it is observed and characterized for the first time the formation of magnesium phosphate crystals during the IVT reaction. Strategies informed by thermodynamic modeling are developed to prevent this undesired crystallization during fed‐batch IVT. Finally, co‐transcriptional capping is incorporated into the model‐based optimization approach and a strategy to maximize RNA formation is developed while maintaining a high level of 5‐prime cap incorporation and minimizing the use of cap analogs.

## Graphical abstract

Using a mathematical model for the enzyme kinetics and solution thermodynamics of the in vitro transcription reaction, new optimized strategies are developed for fed‐batch transcription that can decrease the resource costs of mRNA synthesis while maintaining critical quality attributes for efficacy in vivo.

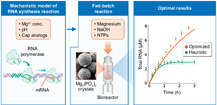

## 1 Introduction

The in vitro transcription (IVT) reaction for synthesis of RNA is a necessary step for the production of a growing number of RNA‐based vaccines and therapeutics. Owing to the breadth of the RNA platform, process engineering strategies to enhance the economic and product quality profile of IVT can have a significant impact on the accessibility of many therapies. IVT is a cell‐free enzymatic polymerization of nucleoside triphosphate (NTP) monomers based on a template DNA sequence and is typically performed as a batch reaction. A key interest in IVT process development is minimizing the use of expensive reagents while maximizing the formation of RNA and quality attributes such as the incorporation of the 5‐prime cap into the RNA product. The 5‐prime cap is a moiety linked to the 5′ end of the RNA molecule that is required for stability and efficacy in vivo. The cap fraction (CF), the fraction of RNA containing a 5‐prime cap, is a key quality attribute of the RNA product. In IVT, this cap is commonly added by the co‐transcriptional incorporation of cap analogs, which mimic the initial nucleotides of the sequence while containing the cap structure. The input materials to IVT can contribute up to 75% of overall RNA manufacturing costs.^[^ ^1^ ^]^ Currently, most of these reagent costs come from polymerase enzymes, DNA templates, and cap analogs. Except for the small proportion of cap analog that is incorporated into RNA, these reagents are not consumed during batch RNA synthesis. For this reason, there is growing interest in performing IVT in a fed‐batch mode to reuse these reagents for the polymerization of additional NTPs.

The transition from batch to fed‐batch IVT greatly increases the number of decision variables for process optimization. Besides the initial reaction conditions, the times and amounts of each reagent feed must also be considered. In addition, practitioners must consider multiple objectives, including maximizing the formation of RNA as well as controlling pH and NTP concentration to desired ranges, which can impact product quality attributes such as the CF. The optimization of fed‐batch IVT is data‐intensive by purely experimental approaches, due to this expanded number of decision variables and their correlated impact on multiple process outputs. Earlier design‐of‐experiment approaches have explored a limited operating space and have not included co‐transcriptional capping.^[^ ^2^ ^]^ Other strategies have relied on iterated fed‐batch experiments to develop a data‐driven understanding of temporal trends in reaction kinetics.^[^ ^3^ ^]^ In addition, previous efforts in fed‐batch IVT have used feedback control strategies, with measurements of pH^[^ ^4^ ^]^ or NTP concentrations,^[^ ^5^ ^,^ ^6^ ^]^ to maintain reaction conditions at desired setpoints. While feedback control methods reduce the number of decision variables, their performance remains dependent on an informed selection of initial conditions and setpoints. In addition, the time delay of offline measurements (such as NTP quantification) can decrease the precision of setpoint tracking.

Model‐based optimization can effectively manage the multivariate input space of IVT and balance the needs for both cost‐effective RNA production and quality of the RNA product. Existing process models for IVT reactions focus on a batch operating mode^[^ ^7^ ^,^ ^8^ ^]^ and cannot be used for fed‐batch reactions, which explore a broader operating space. Notably, it has been repeatedly observed that fed‐batch IVT reactions significantly decrease in rate as the reaction progresses, which is a key barrier to economic performance.^[^ ^4^ ^–^ ^6^ ^]^ A model‐based strategy to address this reaction rate decline requires an improved understanding of the underlying chemistry and physics present in the IVT system.

This work focuses on engineering fed‐batch IVT as a dynamic system with the goal of maximizing RNA production for a given set of DNA, RNA polymerase, and cap analog inputs while achieving a target CF. We develop the first mechanistic process model that describes key trends in fed‐batch IVT such as the decrease in reaction rate at high conversion. Using this model, we compute optimal reaction conditions for tracking NTP setpoints and maximizing the production of RNA using a model DNA sequence that is highly sensitive to salt concentrations. We demonstrate the effectiveness of these methods compared to heuristic strategies in an experimental setup. Finally, we incorporate to our model an expression to predict the CF and design fed‐batch strategies that maximize RNA output per cap analog input while maintaining high (≈90%) CFs.

## 2 Results

### 2.1 Mechanistic Model for Fed‐Batch IVT

**Figure 1:**
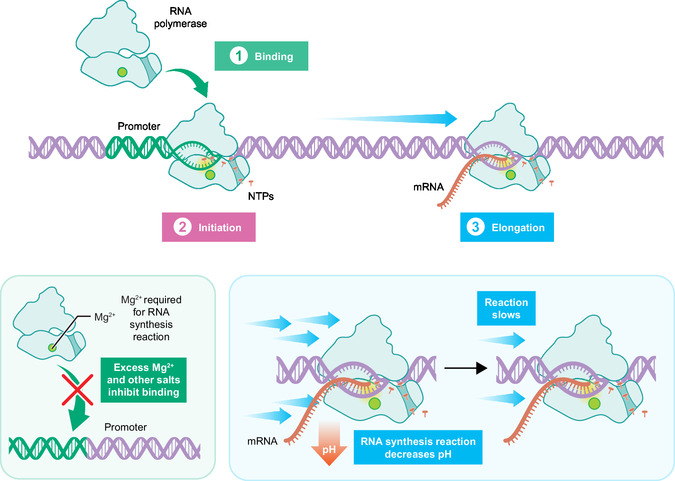
Schematic of the IVT model. RNA is formed by the polymerization of NTPs by DNA and T7 RNA polymerase catalysts. The presence of salts disrupts polymerase‐DNA binding. The transcription reaction decreases the solution pH, which can in turn decrease reaction rates.

A full explanation of the fed‐batch IVT model formulation and parameter estimation strategy is presented in the Supporting Information (Sections S4–5). The fed‐batch IVT model is built upon a batch model presented in an earlier publication.^[^ ^7^ ^]^ Briefly, RNA is synthesized from a DNA template by RNA polymerase, which incorporates NTPs into a growing chain (**Figure** **1**). This process includes steps of binding, initiation, and elongation, and is sensitive to solution conditions such as NTP concentrations and the concentration of the essential Mg cofactor. The model merges a set of differential equations describing enzymatic kinetics with nonlinear algebraic equations describing instantaneous ionic speciation. The key additions required to extend the model to describe fed‐batch IVT relate to the effect of salts and pH on the rate of reaction, as further discussed.

**Figure 2:**
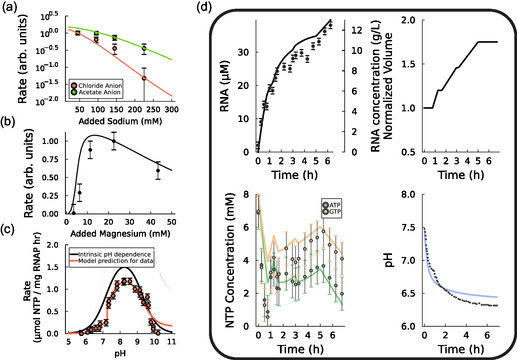
Validation of the mechanistic model. a) IVT reaction rate versus sodium chloride or sodium acetate addition, data from ref. [9]. These data are used for model validation. b) IVT rate versus magnesium acetate addition, data from ref. [9]. These data are used for model validation. c) IVT rate versus pH. Model fit to experimental data^[^ ^13^ ^]^ (red) and the intrinsic pH dependence (Equation 3) recovered from this fitting (black). d) Literature data from a fed‐batch IVT reaction used for model validation,^[^ ^5^ ^]^ showing dynamic trends of RNA concentration, reactor volume, concentration of ATP (yellow) and GTP (green), and pH. In all figures, continuous lines indicate model predictions, while experimental data are reported as points.

Salts, such as the counterions of NTPs added during feeding, can be present in higher concentrations in fed‐batch reactions than in batch reactions. Earlier work has shown that the rate of IVT is highly sensitive to salt addition.^[^ ^9^ ^]^ Our model describes these salt effects by accounting for the effect of ions on polymerase‐DNA binding through a recently derived equation.^[^ ^10^ ^]^ The rate constant for polymerase‐DNA promoter detachment is

(1) $$k_{\mathrm{off}}=k_{\mathrm{off},1M}{\left[\mathrm{salt}\right]}^{n_{\text{salt}}}$$

where $k_{\mathrm{off},1M}$ is a parameter representing the effective binding strength of the polymerase–promoter complex, and the effective salt concentration is

(2) $$\left[\mathrm{salt}\right]=\sum_{i=1}^{N_{\text{ion}}}\omega_{\text{ion},i}[{\mathrm{ion}}_{i}]$$

where $\omega_{\text{ion},i}$ is an ion‐specific parameter representing the relative contribution of each ionic complex (Table S10, Supporting Information) to the effective salt concentration, and $\left[{\mathrm{ion}}_{i}\right]$ is the concentration of a given ionic complex i. This equation sums over all $N_{\text{ion}}$ complexes in the system, which are enumerated in our speciation model. This model can inform the choice of reagents by capturing the differential effect of adding sodium chloride and sodium acetate on IVT kinetics (**Figure** **2**a). In addition, the model effectively predicts the decrease in reaction rate that is experimentally registered at high Mg concentrations due to the highly disruptive effect of Mg$2^{+}$ ions on polymerase‐promoter binding^[^ ^11^ ^]^ (Figure 2b).

In contrast to batch reactions, fed‐batch reactions exhibit non‐negligible dynamic trends in pH which can affect enzyme performance. The solution pH decreases with reaction progression due to the formation of the nucleic acid product. Because the overall drop in pH is related to the total quantity of RNA synthesized relative to the buffering capacity of the solution, fed‐batch reactions can be subject to larger pH changes than typical batch reactions, decreasing by more than one unit. Our model for ionic speciation, which describes the protonation of ions in the IVT solution, was used to calculate the pH as a function of reaction progression. To describe the effect of pH on the reaction rate, rate constants are multiplied by a dimensionless factor $\Gamma_{\mathrm{pH}}$, where

(3) $$\Gamma_{\mathrm{pH}}={\left(1+\frac{\left[H^{+}\right]}{K_{a}}+\frac{K_{b}}{\left[H^{+}\right]}\right)}^{\;\;-1}$$

in keeping with classical literature on enzymatic pH dependence,^[^ ^12^ ^]^ where $K_{a}$ and $K_{b}$ are parameters estimated from data.^[^ ^13^ ^]^ Notably, experimental data describing the effect of pH on IVT rates is convoluted by the salt effects discussed above, as pH is varied experimentally by the addition of ions. Our overall process model was crucial for inferring the intrinsic pH dependence (Equation 3) of IVT from these distorted data (Figure 2c).

We successfully validated the developed IVT model with a vast array of literature data of batch and fed‐batch reactions (Section S6, Supporting Information). The model accurately describes the commonly‐reported reaction rate decline in fed‐batch IVT.^[^ ^4^ ^,^ ^6^ ^]^ The model identifies the accumulation of salts, dilution of catalysts, and the drop in the reaction pH as the primary causes of reaction rate declines in fed‐batch IVT. Notably, the model can also predict the dynamic trends of a fed‐batch reaction with continuous feeding, accurately describing a decrease in pH, nonobvious dynamic trends in NTP concentrations, and a decline in the reaction rate (Figure 2d).

### 2.2 Model‐Based Setpoint Tracking and RNA Yield Optimization

After validation on literature data, the model was used to design fed‐batch IVT protocols. These experiments were performed on a DNA construct that had previously been identified to be especially sensitive to salts.^[^ ^10^ ^]^ To model this increased salt sensitivity, the parameter $k_{\mathrm{off},1M}$ was fit to an increased value of $10^{3.93}$ based on these previous data (Table 10, Supporting Information). In the case of all other sequences modeled in this work, this parameter was held constant at a lower value of $10^{2.93}$. Our initial goal was to produce a maximum quantity of RNA given a fixed input of RNA polymerase and DNA catalyst. In addition, we set out to control NTP concentrations and pH during reactor operation as maintaining these solution conditions within certain bounds is desirable for the purity and quality of the RNA product. The optimization problem solved for this purpose was

(4) $$\begin{array}{c} \min_{F}\;\;\;\nu_{\mathrm{NTP}}\sum_{N\in (A,U,C,G)}\;\sum_{t_{i}\in t_{N}}{\left({\left[\mathrm{NTP}\right]}_{F,t_{i}}-{\left[\mathrm{NTP}\right]}_{sp}\right)}^{\;2}\\ +\nu_{\mathrm{pH}}\sum_{t_{i}\in t_{\mathrm{pH}}}\;{\left({\mathrm{pH}}_{F,t_{i}}-{\mathrm{pH}}_{sp}\right)}^{2}-\nu_{\mathrm{RNA}}{\left[\mathrm{RNA}\right]}_{t=t_{f}} \end{array}$$

where F is a vector representing a set of initial conditions and a feeding policy. In practice, this feeding policy was implemented as set of 9–12 bolus additions containing NTPs, Mg, and NaOH spread over 2–3 h of reaction operation. The terms ${\left[\mathrm{NTP}\right]}_{F,t_{i}}$ and ${\mathrm{pH}}_{F,t_{i}}$ represent the NTP concentration and pH given a policy F at a time $t_{i}$. The terms ${\left[\mathrm{NTP}\right]}_{sp}$ and ${\mathrm{pH}}_{sp}$ represent the setpoint NTP concentration and pH, respectively, while the sets $t_{N}$ and $t_{\text{pH}}$ contain the timepoints at which ${\left[\mathrm{NTP}\right]}_{sp}$ and ${\mathrm{pH}}_{sp}$ were evaluated, respectively. The hyperparameters $\nu_{\mathrm{NTP}}$, $\nu_{\mathrm{pH}}$, and $\nu_{\mathrm{RNA}}$ describe the relative importance of NTP setpoint tracking, pH setpoint tracking, and RNA production to the overall objective function.

**Figure 3:**
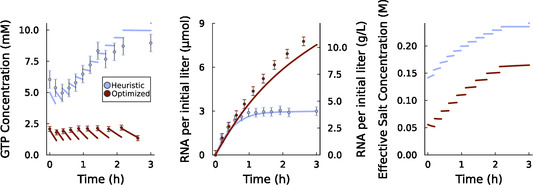
Comparison of the performance of a heuristically designed fed‐batch IVT protocol (blue) with a model‐optimized protocol (red). GTP concentration (left), RNA production (center), and effective salt concentration (right) in reactor as a function of time. Discontinuous jumps represent discrete feeding of solution. The effective salt concentration is defined by (Equation 2).

Using this objective function, we developed an optimized fed‐batch strategy, which is compared to a heuristically developed protocol in **Figure** **3**. The heuristically designed method, which fed NTPs based on initial reaction kinetics, failed to control NTP concentrations to the initial condition of 5 mM each. In addition, the reaction rate of this heuristic protocol decreased rapidly and vanished after the formation of 3 μM RNA (4.3 g L^−1^). This behavior was captured by model predictions. Our model indicated that controlling the concentration of NTPs to a lower setpoint of 2 mM would help to prevent a decline in the reaction rate. In addition to changes in the dynamic feeding of NTPs, our optimization approach changed both the initial and fed quantity of Mg and changed the quantity and formulation of buffer added to the reaction. The optimized method achieved setpoint tracking of NTPs at the 2 mM target, indicating that the model correctly estimated trends in the reaction rate as a function of reaction progression. In addition, the optimized fed‐batch scheme was able to produce greater quantities of RNA, since the rate of reaction did not decay as quickly as in the heuristically designed experiment. After the formation of 7 μM RNA (10 g L^−1^), the optimized approach maintained a reaction rate of 33% of the initial rate.

The increased yield and reaction rates of our optimized protocol are primarily due to a reduction in the effective salt concentration (Equation 2). Our process optimization, which calculated the optimal fed‐batch policy while accounting for the high salt‐sensitivity of the sequence used in this work, had the net effect of decreasing the effective salt concentration by roughly 100 mM (Figure 3C). This result validates our model‐based hypothesis that maintaining low salt concentrations is a key tool for optimization of fed‐batch IVT.

### 2.3 Avoiding Magnesium Phosphate Precipitation with Model‐Based Strategies

**Figure 4:**
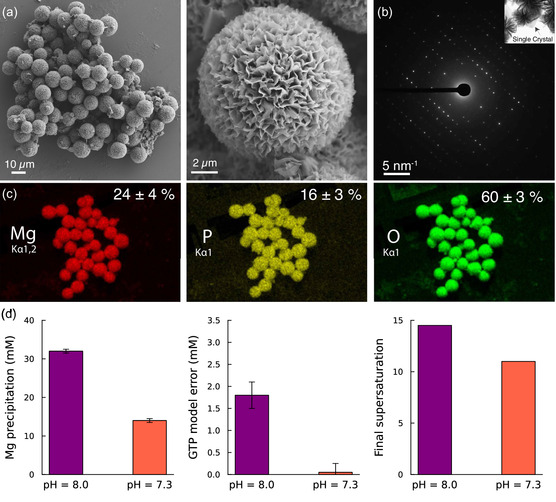
Characterization of magnesium phosphate precipitation during IVT. a) SEM images of magnesium phosphate particles. b) Selected area electron diffraction pattern obtained from a single‐crystal nanosheet of magnesium phosphate. c) Elemental mapping of the particles along with corresponding atomic percentages determined by energy‐dispersive X‐ray spectroscopy. d) Total quantity of magnesium precipitated (left), model‐plant mismatch of final GTP concentration (center), and final predicted supersaturation (right) of high‐pH and low‐pH strategies for fed‐batch operation. The low‐pH strategy corresponds to the optimized process shown in Figure 3.

During the development of the optimized strategy described above, the formation of a solid precipitate was visually observed during reaction operation and characterized using electron microscopy and diffraction (**Figure** **4**a–c). Scanning electron microcopy (SEM) images showed globular microsponges characteristic of crystalline material. Elemental mapping indicated that these structures consisted of magnesium, phosphorous, and oxygen at ratios consistent with the molecular formula of anhydrous ${\text{Mg}}_{3}$(${{\text{PO}}_{4}}_{2}$). The diffraction pattern of a single nano‐sheet matched with the unit cell for an anhydrous magnesium phosphate polymorph (Section S8, Supporting Information).

This observation coincided with a mismatch between model predictions and experimental data that indicated that this precipitation slowed the IVT reaction (Figure 4d). The final concentration of magnesium in the solution was found to be significantly lower than expected, suggesting that this precipitation consumed the majority of magnesium in the reactor. Additionally, it was observed that model predictions for NTP concentrations diverged from experimental measurements after roughly one hour of reaction operation, implying that some phenomenon not captured by the model decreased the reaction rate at late timepoints (Figure 4d, Section S7, Supporting Information). It was hypothesized that the precipitation of magnesium phosphate during the reaction had a negative effect on reaction rates, possibly by decreasing the concentration of magnesium in the solution.

In order to implement the optimized strategy demonstrated above, it was necessary to design the IVT system such that this precipitation would not substantially impact the reaction. Using our speciation model, we attempted to design a reaction strategy which would decrease the thermodynamic driving force of magnesium phosphate precipitation. For this compound, the thermodynamic supersaturation σ was written as

(5) $$\sigma =\ln\;\left(\frac{{\left[{\mathrm{Mg}}^{2+}\right]}^{3}{\left[{\mathrm{PO}}_{4}^{3-}\right]}^{2}}{K_{sp,{\mathrm{Mg}}_{3}{\left({\mathrm{PO}}_{4}\right)}_{2}}}\right)$$

where $K_{sp,{\mathrm{Mg}}_{3}{\left({\mathrm{PO}}_{4}\right)}_{2}}$ is the solubility product of the magnesium phosphate solid. Using our process model, we identified a strategy to decrease this driving force without sacrificing other areas of reaction performance. Since the protonated form of phosphate competes with free phosphate ion, decreasing the pH of the reaction was predicted to decrease the supersaturation. We adjusted our optimal control formulation (Equation 5) from targeting a pH of 8.0 over the entire course of reaction to targeting a setpoint over of 7.3 at the end of the process. In addition, the feeding of magnesium into the reaction was decreased slightly to decrease the free magnesium concentration. These changes decreased the final supersaturation by roughly 25%, assuming no magnesium phosphate precipitation. Experimental implementation of this low‐pH strategy showed that the precipitation of magnesium appeared only at later stages of the reaction and was significantly less than the case of the high‐pH strategy (Figure 4d). In addition, the mismatch between model predictions and experimental NTP concentrations effectively disappeared. This indicates that magnesium phosphate precipitation was the driving source of model‐plant mismatch. This low‐pH strategy was used in the optimized method shown in the earlier section (Figure 3).

### 2.4 Model‐Based Optimization of Co‐Transcriptional Capping

**Figure 5:**
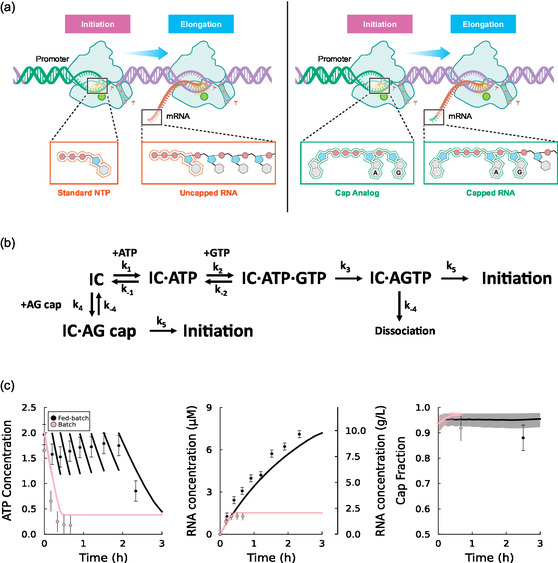
Model‐based optimization of co‐transcriptional capping for IVT. a) Conceptual illustration describing dependence of RNA capping on the first nucleotide added to the RNA sequence. b) Mechanism of competition between trinucleoside AG cap analog and conventional NTP monomers, showing the two competing pathways by which the initiation complex (IC) can incorporate the initial AG nucleotides to the sequence. c) Batch and fed‐batch reactions using co‐transcriptional capping, showing dynamic trends in ATP, RNA, and the CF. The shaded areas for the CF are 95% prediction intervals calculated by Monte Carlo sampling from the parameter covariance matrix.

The fraction of product RNA containing a 5‐prime cap, a key quality attribute of IVT, is dependent on competition between NTPs and cap analogs (**Figure** **5**a). This CF can be maximized for a given cap analog input by dynamically controlling the concentration of NTPs during the reaction. To optimize this process, we developed a mechanistic model to predict the RNA CF as a function of solution conditions. This quasi‐steady‐state kinetic model described the competition between trinucleoside AG cap analogs and NTP monomers (Figure 5b). In this model, the first two base pairs of the RNA sequence can be formed by either the two‐step addition of ATP and GTP, or the one‐step addition of an AG cap analog. Using this kinetic model, an equation for the instantaneous cap fraction (${\text{CF}}_{i}$), or the instantaneous proportion of transcribed RNA containing a five‐prime cap, was derived (Section 2, Supporting Information),

(6) $${\mathrm{CF}}_{i}=\frac{\left[\mathrm{AG}\;\mathrm{cap}\right]}{\left[\mathrm{AG}\;\mathrm{cap}\right]+\frac{\lambda \left[\mathrm{ATP}][\mathrm{GTP}\right]}{1+\frac{\left[\mathrm{GTP}\right]}{\theta }}}$$

where

(7) $$\theta =\frac{k_{-1}(k_{3}+k_{-2})}{k_{2}k_{3}},\;\lambda =\frac{k_{1}}{k_{4}\theta }$$

based on the rate constants shown in Figure 5b. As experiments to quantify CFs are costly and imprecise, a rational design of experiments to identify these parameters was necessary. We used a D‐optimal criterion to develop two experimental batch protocols to identify λ and θ (**Table** **1**). This process used a set of initial estimates gathered from previously published data on an analogous chemical system (Section S3, Supporting Information). The decision variables in this optimization were the initial ATP, GTP, and AG cap concentrations. Intuitively, the first of these batch protocols used a high GTP concentration, while the second used a very low GTP concentration. Maximum likelihood estimation was used to estimate the parameters λ and θ as 25–150 M^−1^ and 0.5–20 mM, respectively.

**Table 1:** Protocol of batch experiments used to calibrate parameters of capping model, including the CF expected based on initial parameter estimates and experimentally measured.

ATP [mM]	GTP [mM]	AG cap [mM]	CF [initial params]	CF [experimental]
3.6	7	0.3	0.48	0.47±0.10
2	0.25	0.05	0.50	0.83±0.05

Using an NTP setpoint of 2 mM, we designed a fed‐batch IVT protocol to maximize RNA production while maintaining a target CF of at least 90%. Our model indicated that the target CF could be achieved by adding only 2 mM of AG cap analog (Figure 5c). To validate our capping model in this regime, a batch experiment was performed using 2 mM of each NTP and AG cap analog. The CF was measured to be 0.92 ± 0.05, slightly but not significantly lower than model predictions of 0.97 ± 0.02. In addition, this model was validated on literature data of a fed‐batch reaction including the use of an AG cap analog^[^ ^3^ ^]^ (Section S6.7, Supporting Information). This validation indicated that the capping model was generally useful for describing trends in data and could be used for designing fed‐batch reactions. Using these setpoints, we developed an optimized fed‐batch protocol that was predicted to yield RNA with a CF between 0.92 and 0.97. Compared to batch operation, the optimized fed‐batch implementation produced four times more RNA, while maintaining a similar CF (0.88 ± 0.05) that was within the range of experimental uncertainty of our target (Figure 5c).

## 3 Discussion

In the context of RNA manufacturing, practitioners seek to optimize the IVT reaction to maximize the production and CF of RNA while minimizing the use of expensive reagents. While fed‐batch reactions are a promising tool for achieving these results, the multivariate nature of this engineering problem makes optimization challenging. In this work, we used a mechanistic process model to design and optimize protocols for fed‐batch IVT. Our results demonstrate the usefulness of this mechanistic approach for achieving set‐point tracking and increasing the output of RNA for a given input of catalysts and cap analogs, which are the key drivers of the cost of goods sold.

A mechanistic approach was critical to understanding and ameliorating the observed decline in reaction rate during fed‐batch operation. Our model includes multiple mechanisms that may cause this decline, including dilution of catalysts, accumulation of salts, and a drop in the pH, and can identify which of these pathways is the primary contributor to an observed reaction decline. Previous work has noted that the construct used in our experiments exhibits an increased sensitivity to salts.^[^ ^10^ ^]^ Heuristic approaches to fed‐batch design exhibited a rapid decline in reaction rate due to the accumulation of these salts (Figure 3). Counterfactual model simulations show that these heuristic approaches would have performed adequately had this construct exhibited a lower salt sensitivity, indicating that accounting for the specific kinetic characteristics of IVT constructs is key in the design of fed‐batch protocols (Section S9, Supporting Information).

Using our mechanistic understanding, the reaction protocol was altered in a number of ways to decrease the overall salt concentration and the salt sensitivity of the reaction system. These changes included modifications to buffer composition, NTP setpoints, magnesium concentrations, and the relative concentration of RNA polymerase and DNA (while keeping overall catalyst costs constant). Decreasing the NTP setpoint and the magnesium cofactor concentration accounted for about half of the total decrease in the effective salt concentration, while replacing the commonly‐used tris‐HCl buffer with a lower concentration of tris base accounted for the other half of the decrease. While previous researchers have experimented with reducing effective salt concentrations by replacing tris‐HCl buffers with tris‐acetate buffers,^[^ ^14^ ^]^ a model‐based approach can go farther by predicting the minimal concentration of buffer required to control reaction pH to a desired window. Our optimized reaction protocol declined in rate at a much slower pace and produced over twice as much RNA as the heuristic strategy.

Our optimization approach successfully controlled NTP concentrations within the range of 1–2 mM during reactor operation. Past efforts in feedback control for fed‐batch IVT have struggled to control NTP concentrations, due to time delays in offline measurements. For example, NTP concentrations in previously studied fed‐batch systems have varied between 1 and 8 mM (Figure 2d). Wide fluctuations in system dynamics can increase batch‐to‐batch variation of quality attributes that depend on NTP concentrations such as the CF. In our work, most of the NTP fluctuation was due to our bolus feeding scheme. Future work using continuous feeding can further reduce NTP variation during IVT.

In addition to identifying optimal reaction strategies, our mechanistic model was key to understanding and overcoming barriers during process development. The speciation model developed in this work was crucial to understanding the effect of process parameters on the thermodynamic driving force of unwanted magnesium phosphate precipitation during the reaction. While magnesium pyrophosphate precipitation during IVT has been previously observed, characterized, and modeled,^[^ ^15^ ^]^ our use of the pyrophosphatase enzyme prevents pyrophosphate accumulation by degrading it to phosphate. This work is the first observation in the research literature of magnesium phosphate precipitation during IVT. As this is the first report of this precipitation occurring during IVT, we could not rely on data‐driven solutions to this problem. Our first‐principles approach allowed us to eliminate the deleterious effects of this precipitation with minimal experimental iteration (Figure 4).

One of the most promising applications of fed‐batch IVT is in efficiently using cap analogs by dynamically controlling NTP concentrations during the reaction. Using an optimally selected set of batch experiments, we incorporated an expression to predict capping into our overall process model. This allowed us to predicatively design fed‐batch reactions to produce a maximum amount of RNA per cap analog input, while maintaining the final capping fraction at a desired level (≈90%). This approach can allow practitioners to operate at the desired point on the Pareto frontier of CF and cap analog consumption. For example, our model can also accurately describe the results of fed‐batch reactions which achieve higher (>95%) CFs while consuming three times greater quantities of AG cap analog^[^ ^3^ ^]^ (Section S6.7, Supporting Information). In this case, the CF exhibited a dynamic trend due to dynamics of NTP and cap concentrations, demonstrating the need for a dynamic process model in predicting CFs.

A comparative analysis shows that the key area of improvement over previous work is in reducing the range of dynamic variation of NTP concentrations, which is useful for controlling product quality attributes such as CFs (Section S10, Supporting Information). In addition, a composite metric of reaction productivity indicates that this work uses the polymerase enzyme slightly more efficiently than previous work (despite the salt‐sensitivity of this sequence), which we attribute to the model‐based control of optimal reaction conditions such as magnesium concentrations throughout the process. As the exact objective function that practitioners are subject to may evolve with time, this model‐based approach is a flexible platform to achive diverse and multivariate goals. While this work focuses on optimizing the synthesis of a single RNA sequence, this model‐based optimization workflow is generalizable to any sequence. Applying this approach to a new sequence would require an understanding of the kinetic parameters of the sequence, which could be estimated using a single round of targeted experiments.^[^ ^10^ ^]^ In addition, while this work focused only on the CF, there are a number of RNA quality attributes that could potentially be incorporated into this model‐based optimization strategy, including double‐stranded RNA concentrations and the formation of RNA shorter than the desired target sequence. Including these quality attributes would require the development of kinetic models detailing the mechanisms and input–output relationships of the formation of these byproducts. Finally, the model presented in this work can be extended to describe the operation of a continuous stirred tank reactor (CSTR) by adding mathematical terms describing the continuous removal of liquid from the reactor. However, it is possible that additional physical phenomena, such as the degradation of biomolecule catalysts and viscosity induced by high RNA concentrations,^[^ ^14^ ^]^ may need to be added to describe the behavior of CSTRs with residence times longer than the reaction times studied in this work. For the first time, we have developed a model‐based approach for the optimization of fed‐batch IVT reactions that can enable dynamic control of NTP concentrations and CFs while maximizing the synthesis of RNA for DNA sequences with diverse kinetic characteristics. We have also used our modeling approach to control the pH trajectory, which can enable control of additional quality attributes of RNA in future work.

## Supporting Information

An electronic supporting information detailing methods, derivations, and model validation is included with this work.^[^ ^3^ ^,^ ^11^ ^,^ ^14^ ^,^ ^16^, ^17^, ^18^, ^19^, ^20^, ^21^, ^22^, ^23^, ^24^, ^25^, ^26^ ^–^ ^27^ ^]^ All data and code used in this work will be made publicly available upon acceptance of the manuscript.

## Conflict of Interest

The authors declare no conflict of interest.

## Author Contributions

**Nathan** **Merica Stover**: conceptualization (lead); data curation (lead); formal analysis (lead); investigation (lead); methodology (lead); resources (lead); software (lead); supervision (lead); validation (lead); visualization (lead); writing—original draft (lead); writing—review and editing (lead). **Soroush Ahmadi**: conceptualization (supporting); formal analysis (supporting); investigation (supporting); methodology (supporting); resources (supporting); visualization (supporting); writing—original draft (supporting). **Jacob Rosenfeld**: conceptualization (supporting); investigation (supporting); methodology (supporting); resources (supporting). **Francesco Destro**: conceptualization (supporting); formal analysis (supporting); methodology (supporting); writing—original draft (supporting); writing—review and editing (supporting). **Allan S. Myerson**: conceptualization (supporting); funding acquisition (supporting); methodology (supporting); project administration (supporting); supervision (supporting). **Richard D. Braatz**: conceptualization (supporting); funding acquisition (lead); supervision (lead); writing—review and editing (supporting).

## Supporting information

Supplementary Material

## Data Availability

The data that support the findings of this study are openly available in Github at https://github.com/nathanmstover/fedbatchIVT, reference number 0.

## References

[1] Z. Kis , K. Tak , D. Ibrahim , S. Daniel , D. van de Berg , M. M. Papathanasiou , B. Chachuat , C. Kontoravdi , N. Shah , Comp. Aided Chem. Eng. 2022, 49, 2167.

[2] L. Guo , Z. Liu , S. Song , W. Yao , M. Yang , G. Chen , Biochem. Eng. J. 2024, 210, 109412.

[3] J. Elich , A. E. Rabideau , M. Shamashkin , R. Philpot , B. Fritz , P. Wojciechowski , Fed‐Batch in Vitro Transcription Process 2022.

[4] J. A. Kern , R. H. Davis , Biotechnol. Prog. 1999, 15, 174. 10194392 10.1021/bp990008g

[5] J. Skok , P. Megušar , T. Vodopivec , D. Pregeljc , N. Mencin , M. Korenč , A. Krušič , A. M. Celjar , N. Pavlin , J. Krušič , M. Mueller , K. McHugh , A. Štrancar , R. Sekirnik , Chemie Ingenieur Technik 2022, 94, 1928.

[6] D. Pregeljc , J. Skok , T. Vodopivec , N. Mencin , A. Krušič , J. Ličen , K. v. Nemec , A. Štrancar , R. Sekirnik , Biotechnol. Bioeng. 2023, 120, 737. 36471904 10.1002/bit.28299

[7] N. M. Stover , K. Ganko , R. D. Braatz , Biotechnol. Bioeng. 2024, 121, 2636. 38695152 10.1002/bit.28699

[8] D. van de Berg , Z. Kis , C. F. Behmer , K. Samnuan , A. K. Blakney , C. Kontoravdi , R. Shattock , N. Shah , Npj Vaccines 2021, 6, 1. 33927197 10.1038/s41541-021-00322-7 PMC8085199

[9] J. A. Kern , R. H. Davis , Biotechnol. Prog. 1997, 13, 747. 9413132 10.1021/bp970094p

[10] N. M. Stover , M. D. Bock , J. Chen , J. Rosenfeld , M. D. C. P. Royo , A. S. Myerson , R. D. Braatz 2025.

[11] T. Łoziński , K. L. Wierzchowski , Acta Biochim. Pol. 2009, 56, 695. 19898692

[12] R. A. Alberty , V. Massey , Biochim. Biophys. Acta 1954, 13, 347. 13140346 10.1016/0006-3002(54)90340-6

[13] P. A. Osumi‐Davis , N. Sreerama , D. B. Volkin , C. R. Middaugh , R. W. Woody , A.‐Y. M. Woody , J. Mol. Biol. 1994, 237, 5. 8133519 10.1006/jmbi.1994.1205

[14] J. Boman , T. Marušič , T. V. Seravalli , J. Skok , F. Pettersson , K. v. Nemec , H. Widmark , R. Sekirnik , Biotechnol. Bioeng. 2024, 121, 3415. 39014536 10.1002/bit.28806

[15] S. Akama , M. Yamamura , T. Kigawa , Biophys. J. 2012, 102, 221. 22339858 10.1016/j.bpj.2011.12.014 PMC3260666

[16] E. N. Welbourne , K. A. Loveday , A. Nair , E. Nourafkan , J. Qu , K. Cook , Z. Kis , M. J. Dickman , Front. Mol. Biosci. 2024, 11, 1250833. 38516194 10.3389/fmolb.2024.1250833 PMC10955092

[17] D. C. Liu , J. Nocedal , Math. Program. 1989, 45, 503.

[18] H. Krakauer , Biopolymers 1971, 10, 2459. 5126519 10.1002/bip.360101209

[19] R. M. Smith , A. E. Martell , Critical Stability Constants, Springer US, Boston, MA 1976.

[20] J. S. Young , W. F. Ramirez , R. H. Davis , Biotechnol. Bioeng. 1997, 56, 210. 18636626 10.1002/(SICI)1097-0290(19971020)56:2<210::AID-BIT10>3.0.CO;2-K

[21] R. A. Alberty , R. N. Goldberg , Biochemistry 1992, 31, 10610. 1420176 10.1021/bi00158a025

[22] T. Günther , J. Vormann , P. Konstanczak , A. Schäfer , Biochim. Biophys. Acta 1994, 1192, 281. 8018709 10.1016/0005-2736(94)90129-5

[23] M. Maslak , C. T. Martin , Biochemistry 1994, 33, 6918. 7911327 10.1021/bi00188a022

[24] G.‐Q. Tang , V. S. Anand , S. S. Patel , J. Mol. Biol. 2011, 405, 666. 21035457 10.1016/j.jmb.2010.10.020 PMC3053063

[25] H. R. Koh , R. Roy , M. Sorokina , G.‐Q. Tang , D. Nandakumar , S. S. Patel , T. Ha , Mol. Cell 2018, 70, 695. 29775583 10.1016/j.molcel.2018.04.018 PMC5983381

[26] A. Nord , P. Kierkegaard , Acta Chem. Scand. 1968, 22, 1466.

[27] M. Klinger , A. Jäger , J. Appl. Crystallogr. 2015, 48, 2012. 26664349 10.1107/S1600576715017252 PMC4665667

