# Supplementary material for: Model‐Based Optimization of Fed‐Batch In Vitro Transcription
Source: Chembiochem. 2025 Oct 9;26(21):e202500485. doi: 10.1002/cbic.202500485 (PMC12596937; doi:10.1002/cbic.202500485)
Supplement: Supplementary file 1 — Supplementary Material [file CBIC-26-e202500485-s001.pdf]

Supporting Information: Model-Based Optimization of Fed-Batch In Vitro Transcription

Nathan Merica Stover, Soroush Ahmadi, Jacob Rosenfeld, Francesco Destro, Allan S. Myerson, Richard D. Braatz*

stover@mit.edu, braatz@mit.edu
* Corresponding author

Department of Chemical Engineering, Massachusetts Institute of Technology, 77 Massachusetts Avenue, Cambridge, MA 02139, USA

Keywords: mRNA synthesis, in vitro transcription, optimal control, co-transcriptional capping.

Contents

1 Methods **3**
1.1 In vitro transcription reactions . . . 3
1.2 Fed-batch protocols . . . 3
1.2.1 Heuristic process (Figure 3 of the main text) . . . 3
1.2.2 High-pH process (Figure 4 of the main text and Figure 17 of the SI) . . 4
1.2.3 Optimized low-pH process (Figure 3 of the main text, Figure 5 of the main text, and Figure 17 of the SI) . . . 5
1.2.4 Optimized process including capping (Figure 5 of the main text) . . . 6
1.3 Dynamic optimization . . . 6
1.4 Model-based design of experiments . . . 7

2 Derivation of Laws for Cap Fraction **7**
2.1 Binucleoside cap analogs . . . 7
2.2 Trinucleoside cap analogs . . . 8

3 Estimation of Parameters for Trinucleoside Capping **9**
3.1 Choice of initial parameter estimates . . . 9
3.2 Final Parameter Estimation . . . 9

4 Overall Dynamic Model Formulation **10**

5 Parameter Estimation **13**

6 Additional Model Validation **14**
6.1 Kern and Davis (1997) [9] . . . 14
6.2 Samnuan et al. (2022) [21] . . . 16
6.3 Pregljc et al. (2023) [2] . . . 17
6.4 Kern and Davis (1999) [6] . . . 20

6.5 Skok et al. (2022) [22] 22
6.6 Elich et al. [4] (2022) 23
6.7 Boman et al. (2024) 23

7 Dynamics of Magnesium Phosphate Precipitation 24

8 Characterization of Magnesium Phosphate Precipitate 26

9 Counterfactual Simulation of Heuristic Process Assuming a Lower Salt Sensitivity 26

10 Comparison with Previous Experimental Results 28

1 Methods

1.1 In vitro transcription reactions

All reaction materials were acquired from Hongene Biotech, other than magnesium acetate and tris, which were acquired from Thermo Fisher. NTPs used in this work were buffered using a tris counter-ion to a pH of 7.6. Transcription reactions were assembled at volumes between 50–100 μL and incubated at 37°C. The linearized DNA plasmid used for transcription encoded for the COVID spike protein and contained 1065, 801, 1316, and 1061 A, U, C, and G nucleotides respectively. The DNA molecule itself consisted of 6082 base pairs. Bolus additions of feed were added to this reaction during operation. Aliquots of 6 μL were periodically removed and quenched in 60 μL of 50 mM EDTA. To measure solution magnesium concentrations, aliquots were collected from the reaction after brief centrifuging to separate the solution from magnesium phosphate solids. These quenched samples were further diluted 36-fold (for a total dilution of 400-fold) and analyzed with the HPLC method of Welbourne et al. [1] to quantify the concentrations of the four NTPs. The concentration of magnesium in diluted aliquots was measured with inductively coupled plasma mass spectrometry (ICP-MS). The pH of the reaction solution was directly measured with an Orion™ pH electrode. The cap fraction of RNA product was measured using the method applied to fed-batch IVT by [2]. We use the same materials for this assay as these previous researchers.

The "heuristic reaction" shown in Figure 3 of the main text was developed by measuring the initial rate of reaction in a batch context. A discrete feeding strategy of NTPs, Mg, and NaOH was developed to replenish NTPs in this mixture, maintain the pH, and maintain the initial Mg/NTP ratio assuming the reaction would continue at the initial rate.

All batch and fed-batch experiments in this work, including experiments for capping, were performed with three reaction replicates. Error bars in our experimental data represent the standard deviation of results of these three replicates.

1.2 Fed-batch protocols

In all of these cases, 6μL of fluid was removed at the initial time point and at each feeding time point for analytics. The model predictions applied to these data in the main text of this work factor in this removal of fluid.

1.2.1 Heuristic process (Figure 3 of the main text)

This reaction was assembled at an initial volume of 90 μL containing 5 mM of each NTP, no cap analog, 21.1 mM magnesium acetate, 13.9 nM linearized DNA plasmid, 160 nM RNA polymerase, 6 U/mL inorganic pyrophosphatase, 400 U/mL RNAase inhibitor, 40 mM Tris-HCl buffer (pH 8.0), 2 mM spermidine, and 10 mM DTT. The initial reaction mixure was adjusted to a pH of 8.1 by the addition of NaOH. Feeding was performed based on the following tables.

Table 1: Heuristic process: composition of feed stock solution

Species	Concentration (mM)
ATP	47.2
UTP	37.3
CTP	56.5
GTP	47.0
$MgOAc_2$	86.2
NaOH	472

Table 2: Heuristic process: Timing and volume of feeding

Volume (μL)	Time (min)
3.18	10.8
2.96	22.2
2.76	34.2
2.56	46.2
2.35	58.8
2.16	72.0
1.97	85.8
1.79	99.6
1.62	115.2
1.45	130.8

1.2.2 High-pH process (Figure 4 of the main text and Figure 17 of the SI)

This reaction was assembled at an initial volume of 90 μL containing 2 mM of each NTP, no cap analog, 15 mM magnesium acetate, 20 nM linearized DNA plasmid, 100 nM RNA polymerase, 6 U/mL inorganic pyrophosphatase, 400 U/mL RNAase inhibitor, 24.6 mM of tris base, 2 mM spermidine, and 10 mM DTT. Feeding was performed based on the following tables.

Table 3: High-pH process: composition of feed stock solution

Species	Concentration (mM)
ATP	42.6
UTP	32.7
CTP	51.9
GTP	42.5
$MgOAc_2$	237
NaOH	413

Table 4: High-pH process: Timing and volume of feeding

Volume (μL)	Time (min)
2.62	11.4
2.44	22.8
2.26	34.8
2.09	47.4
1.93	61.2
1.76	75.6
1.61	91.2
1.45	108
1.30	126.6

1.2.3 Optimized low-pH process (Figure 3 of the main text, Figure 5 of the main text, and Figure 17 of the SI)

This reaction was assembled at an initial volume of 90 μL containing 2 mM of each NTP, no cap analog, 15 mM magnesium acetate, 20 nM linearized DNA plasmid, 100 nM RNA polymerase, 6 U/mL inorganic pyrophosphatase, 400 U/mL RNAase inhibitor, 24.6 mM of tris base, 2 mM spermidine, and 10 mM DTT. Feeding was performed based on the following tables.

Table 5: Optimized low-pH process: composition of feed stock solution

Species	Concentration (mM)
ATP	30.7
UTP	23.5
CTP	37.5
GTP	30.63
$MgOAc_2$	151
NaOH	69.0

Table 6: Optimized low-pH process: Timing and volume of feeding

Volume (μL)	Time (min)
2.00	12.0
1.86	24.0
1.73	37.2
1.60	51.6
1.46	67.2
1.34	85.2
1.21	105.0
1.09	127.8

1.2.4 Optimized process including capping (Figure 5 of the main text)

This reaction was assembled at an initial volume of 90 μL containing 2 mM of each NTP, 2 mM of an AG cap analog, 15 mM magnesium acetate, 20 nM linearized DNA plasmid, 100 nM RNA polymerase, 6 U/mL inorganic pyrophosphatase, 400 U/mL RNAase inhibitor, 24.6 mM of tris base, 2 mM spermidine, and 10 mM DTT. Feeding was performed based on the following tables.

Table 7: Optimized process (including capping): composition of feed stock solution

Species	Concentration (mM)
ATP	36.9
UTP	28.3
CTP	45.0
GTP	36.8
$MgOAc_2$	182
NaOH	82.9

Table 8: Optimized process (including capping): Timing and volume of feeding

Volume (μL)	Time (min)
2.00	12
1.86	24
1.73	37.2
1.59	51.6
1.46	67.2
1.33	85.2
1.21	105
1.09	127.8

1.3 Dynamic optimization

The optimal trajectory of feeding of NTPs, Mg, and NaOH (for pH control) was computed by solving the optimization problem formulated in equation 4 of the main text. It was found that this optimization problem, which involved $6n_t$ decision variables, where n_t is the number of time intervals used, could be simplified with virtually no reduction in optimality by assuming that NTPs, Mg, and NaOH were added to the reaction in a constant ratio regardless of the time of feeding. This approach additionally simplified experimental implementation by only requiring addition of a single feeding stock solution. The number of decision variables was further reduced by recognizing that the four-dimensional set of reactor NTP concentrations would only dynamically vary on a two-dimensional manifold during reaction operation, where the reduced dimensions correspond to reaction stoichiometry and dilution. In this reformulated optimization, the n_t+3 decision variables represented the relative ratios of NTP, Mg, and NaOH in a feeding stock solution as well as a discretized profile of feeding quantity. To experimentally implement this strategy, the continuous feeding profile was approximated as a series of discrete additions of a feeding stock solution. In addition to decision variables for feeding, a set of decision variables for the initial magnesium and tris buffer conditions were included in this optimization. Control vector parameterization was used to formulate this optimization

problem. Local gradient-based optimization was carried out with L-BFGS optimization using the ForwardDiff.jl and NLopt.jl packages in Julia to compute model output sensitivities to parameters and use those sensitivities in gradient-based optimizers, respectively [3]. To generate the optimal strategy presented in Figure 3 of the main text, this optimization strategy was paired with a number of changes based on mechanistic knowledge of the system. Based on model predictions that reactions with higher DNA concentrations have a decreased sensitivity to salt effects, we adjusted the concentrations of the DNA and RNA polymerase catalysts to include additional DNA while roughly maintaining overall catalyst costs (corresponding to a 40% increase and a 25% decrease in DNA and RNA polymerase concentrations, respectively). In addition, based on our understanding that chloride ions disrupt polymerase-DNA binding, we removed the tris HCl buffer commonly used in IVT, replacing it with a tris base.

1.4 Model-based design of experiments

Due to the difficulty and experimental variance of the cap fraction measurements used in this work, it was necessary to pre-select reaction conditions to allow for minimal experimentation. Model-based design of experiments was used to best estimate the parameters λ and θ. Given a hypothesized parameter set $\hat{p}$ and a prior covariance matrix $\mathrm{cov}(\hat{p})$, experimental points x were chosen by solving the optimization

$$\min_x [S(x,\hat{p})^\top V_y^{-1} S(x,\hat{p})) + \mathrm{cov}(\hat{p})^{-1}]^{-1} \tag{1}$$

where $S(x,\hat{p})$ is the sensitivity matrix of the experimental points x with respect to estimated parameters $\hat{p}$ and V_y is the diagonal error covariance matrix of experimental outputs. Local gradient-based optimization was carried out with L-BFGS optimization using the ForwardDiff.jl and NLopt.jl packages in Julia to compute model output sensitivities to parameters and use those sensitivities in gradient-based optimization, respectively.

2 Derivation of Laws for Cap Fraction

The results of the co-transcriptional capping process are driven by competition between cap analogs and standard NTPs. This section derives the quantitative expressions used for capping fraction in this work for both incorporation of binucleoside and trinucleoside capping analogs (Figure 1 A and B, respectively). The processes shown below are approximated as operating in a quasi-steady state as the timescales of individual steps are much shorter than the overall reaction timescale used in this work.

2.1 Binucleoside cap analogs

By applying a quasi-steady state assumption, the concentrations of each of the complexes in Figure 1A can be related to the concentration of the initiation complex (IC) by

$$[\mathrm{IC}\cdot\mathrm{GTP}] = \frac{k_1[\mathrm{GTP}][\mathrm{IC}]}{k_2 + k_{-1}}, \qquad [\mathrm{IC}\cdot\mathrm{G\ cap}] = \frac{k_3[\mathrm{G\ cap}][\mathrm{IC}]}{k_2 + k_{-3}}. \tag{2}$$

The cap fraction is defined as the ratio of capped RNA initation events to the total initiation events,

$$\mathrm{CF}_i = \frac{k_2[\mathrm{IC}\cdot\mathrm{G\ cap}]}{k_2[\mathrm{IC}\cdot\mathrm{G\ cap}] + k_2[\mathrm{IC}\cdot\mathrm{GTP}]} = \frac{[\mathrm{G\ cap}]}{[\mathrm{G\ cap}] + \gamma_{bi}[\mathrm{GTP}]}, \tag{3}$$

where

$$\gamma_{bi} = \frac{k_1(k_2 + k_{-3})}{k_3(k_2 + k_{-1})}. \tag{4}$$

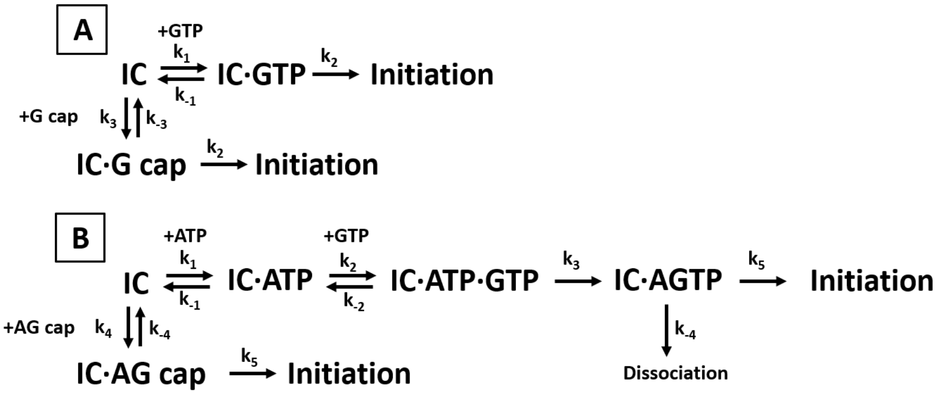

Figure 1: Schematic mechanisms describing competition between cap analogs and NTPs. (A) In the case of binucleoside G cap analogs (eg. anti-reverse capping analog, ARCA), the single-step addition of a GTP analog to the initiation complex (IC) competes with the single-step addition of GTP for the first nucleotide site. (B) In the case of trinucleoside AG cap analogs (eg. CleanCap), ATP-GTP addition requires two kinetic steps, while the addition of cap analog requires one kinetic step.

Due to the chemical similarity of GTP and its corresponding cap analog, a reasonable prior for this parameter is 1.

2.2 Trinucleoside cap analogs

We again assume a quasi-steady state for this system (Figure 1B). By using mass balances, the concentrations of IC · ATP · GTP and IC · ATP can be related using a Michaelis-Menten constant,

$$[\text{IC} \cdot \text{ATP} \cdot \text{GTP}] = \frac{k_2[\text{IC} \cdot \text{ATP}][\text{GTP}]}{k_3 + k_{-2}} = \frac{[\text{IC} \cdot \text{ATP}][\text{GTP}]}{K_{M,\text{GTP}}} \tag{5}$$

Secondly, the concentration of IC and IC · AG cap can be related using a Michaelis-Menten constant,

$$[\text{IC} \cdot \text{AG cap}] = \frac{k_4[\text{IC}][\text{AG cap}]}{k_5 + k_{-4}} = \frac{[\text{IC}][\text{AG cap}]}{K_{M,\text{AG cap}}}. \tag{6}$$

Performing a mass balance for IC · AGTP, considering that each process is first order and independent of NTP concentration, gives that

$$[\text{IC} \cdot \text{AGTP}] = \frac{k_3}{k_5 + k_{-4}}[\text{IC} \cdot \text{ATP} \cdot \text{GTP}]. \tag{7}$$

The mass balance for IC · ATP is

$$\frac{d[\text{IC} \cdot \text{ATP}]}{dt} = 0 = k_1[\text{IC}][\text{ATP}] - k_{-1}[\text{IC} \cdot \text{ATP}] - k_2[\text{IC} \cdot \text{ATP}][\text{GTP}] + k_{-2}[\text{IC} \cdot \text{ATP} \cdot \text{GTP}], \tag{8}$$

$$0 = k_1[\text{IC}][\text{ATP}] - k_{-1}[\text{IC} \cdot \text{ATP}] - k_2[\text{IC} \cdot \text{ATP}][\text{GTP}] + \frac{k_{-2}[\text{IC} \cdot \text{ATP}][\text{GTP}]}{K_{M,\text{GTP}}}, \tag{9}$$

$$[\text{IC} \cdot \text{ATP}] = \frac{k_1[\text{IC}][\text{ATP}]}{k_{-1} + (k_2 - \frac{k_{-2}}{K_{M,\text{GTP}}})[\text{GTP}]} \tag{10}$$

The quantity of interest is the fraction of initiation events that are capped,

$$\text{CF}_i = \frac{k_5[\text{IC} \cdot \text{AG cap}]}{k_5[\text{IC} \cdot \text{AG cap}] + k_5[\text{IC} \cdot \text{AGTP}]} = \frac{[\text{AG cap}]}{[\text{AG cap}] + \frac{\gamma[\text{ATP}][\text{GTP}]}{\theta + [\text{GTP}]}} \tag{11}$$

where

$$\gamma = \frac{k_1}{k_4}, \qquad \theta = \frac{k_{-1}(k_3 + k_{-2})}{k_2 k_3}. \tag{12}$$

For the purpose of parameter estimation, the parameters θ and

$$\lambda = \frac{\gamma}{\theta} \tag{13}$$

are estimated to decrease correlation in the parameter covariance matrix.

The above formulations can be used to inform our priors for these parameters. It is difficult to determine γ in the absence of data. However, an approach to develop a prior guess for θ is to observe that

$$\frac{k_3 + k_{-2}}{k_2} \tag{14}$$

has a mechanistic similarity to the parameter K_1 for overall IVT MgNTP dependence that has been estimated in past work. In the absence of any information about the ratio k_{-1}/k_3, a reasonable prior is one, giving the estimate that $\theta \approx K_1 \approx 0.22$ mM.

3 Estimation of Parameters for Trinucleoside Capping

3.1 Choice of initial parameter estimates

In order to design experiments to identify the parameters in our capping model, it was necessary to have an approximate initial estimate of these parameters. We estimated these values based on published data on trinucleoside capping [4]. These data quantified the final cap fraction of RNA product as a function of NTP/AG cap ratios.

While these data did not include all of the conditions needed to fit the process model, they were suitable for a rough estimation. In the following, we make the approximation that our expression for the instantaneous cap fraction is equivalent to the final measured capping fraction of these data ($\mathrm{CF}_i \approx \mathrm{CF}$). In the case that $[\mathrm{GTP}] \gg \theta$,

$$\mathrm{CF}_i = \frac{[\text{AG cap}]}{[\text{AG cap}] + \gamma[\text{ATP}]}, \tag{15}$$

and a plot of 1/CF over [ATP]/[AG cap] would yield a straight line with a slope of γ and an intercept of one (Figure 2A). From experimental data, a reasonable estimate for γ is 0.12. Alternatively, in the case that $[\mathrm{GTP}] \ll \theta$,

$$\mathrm{CF}_i = \frac{[\text{AG cap}]}{[\text{AG cap}] + \frac{\gamma[\text{ATP}]}{\theta}[\text{GTP}]}, \tag{16}$$

for which a plot of 1/CF over [GTP]/[AG cap] would yield a straight line with a slope of $\frac{\gamma[\text{ATP}]}{\theta}$ and an intercept of one.

A reasonable estimate for θ was calculated to be 0.24 mM. This value is close to that estimated by an orthogonal method in the previous section, which gives greater physical confidence in this result.

3.2 Final Parameter Estimation

Using the data shown in the main text (Table 1), maximum likelihood estimation was used to estimate the parameters λ and θ as 25–150 M^{-1} and 0.5–20 mM, respectively. As these estimates are correlated, the parameter uncertainty interval is best depicted using a 95% confidence ellipsoid calculated using the same linearized uncertainty methods used in [5] (Figure 3).

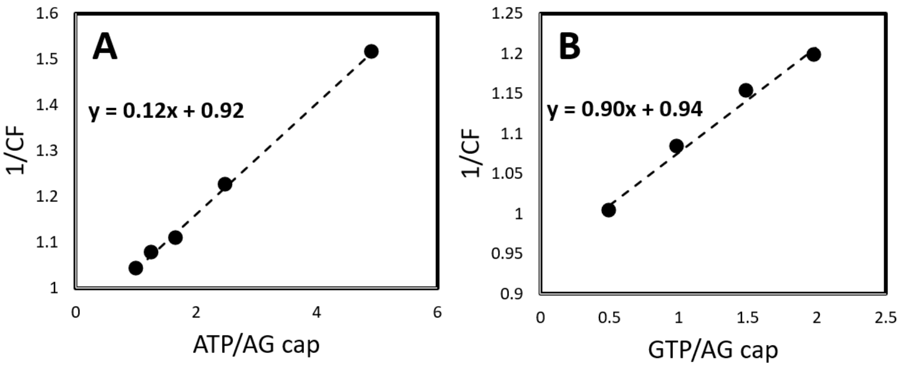

Figure 2: Plots of experimental data for the inverse of the final capping fraction (1/CF) as a function of NTP/AG cap ratios [4].

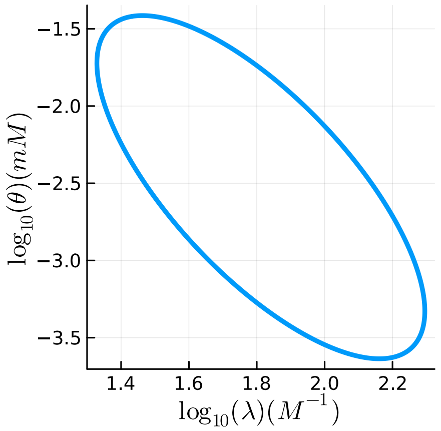

Figure 3: Estimated 95% conference ellipsoid quantifying uncertainty in θ and λ.

4 Overall Dynamic Model Formulation

This section details the model formulation used in this work. The model is an augmented version of a model published in our past work [5]. The model does not explicitly write out the kinetic steps for the formation of magnesium pyrophosphate solids, as the pyrophosphatase enzyme was added to all experimental systems studied in this work (other than that of [6]). Also, pyrophosphate (PPi) is assumed to instantly hydrolyze to orthophosphate (Pi) (in contrast, [6], assumed that this hydrolysis did not take place). This means that the entire system contains only one dynamic process, the transcription reaction itself, which has the overall stoichiometry

$$\mathrm{N_A(ATP) + N_U(UTP) + N_C(CTP) + N_G(GTP) \rightarrow RNA + (N_{all}-1)PPi + (N_{all}-1)H} \quad (17)$$

where

$$\mathrm{N_{all} = N_A + N_U + N_C + N_G} \quad (18)$$

and $\mathrm{N_A}$, $\mathrm{N_U}$, $\mathrm{N_C}$, and $\mathrm{N_G}$ are the numbers of ATP, UTP, CTP, and GTP monomers incorporated into each RNA sequence. This model is formulated to accommodate fed-batch reactions by allowing reactor volume to change over time. The dynamic system is reformulated around a single differential equation: the rate of RNA synthesis in M/hr (R_{tr}). The corresponding state variable is the integral amount of RNA produced in moles (ξ), i.e.,

$$\frac{\mathrm{d}\xi}{\mathrm{d}t} = R_{\mathrm{tr}}V \quad (19)$$

where V is the volume of the reactor. Total concentrations of NTPs, RNA, and the phosphate byproduct are calculated based on this total extent of reaction,

$$V \begin{bmatrix} [\text{ATP}]_{\text{tot}} \\ [\text{UTP}]_{\text{tot}} \\ [\text{CTP}]_{\text{tot}} \\ [\text{GTP}]_{\text{tot}} \\ [\text{RNA}]_{\text{tot}} \\ [\text{Pi}]_{\text{tot}} \end{bmatrix} = \begin{bmatrix} F_{\text{ATP}}(t) \\ F_{\text{UTP}}(t) \\ F_{\text{CTP}}(t) \\ F_{\text{GTP}}(t) \\ 0 \\ 0 \end{bmatrix} + \xi \begin{bmatrix} -\text{N}_{\text{A}} \\ -\text{N}_{\text{U}} \\ -\text{N}_{\text{C}} \\ -\text{N}_{\text{G}} \\ 1 \\ \text{N}_{\text{all}} - 1 \end{bmatrix}, \tag{20}$$

where F_{ATP} is the total quantity, in moles, of ATP added the reaction by both initial conditions and feeding. The rate of transcription is determined by an initiation-elongation model detailed in previous work [7],

$$R_{\text{tr}} = k_I [\text{P} \cdot \text{DNA}_{\text{p}}] \tag{21}$$

where

$$[\text{P} \cdot \text{DNA}_{\text{p}}] = \frac{[\text{P}]_{\text{tot}} + \alpha [\text{DNA}_{\text{p}}]_{\text{tot}} + K_{\text{MD}}}{2\alpha} - \frac{\sqrt{([\text{P}]_{\text{tot}} + \alpha [\text{DNA}_{\text{p}}]_{\text{tot}} + K_{\text{MD}})^2 - 4\alpha [\text{P}]_{\text{tot}} [\text{DNA}_{\text{p}}]_{\text{tot}}}}{2\alpha}, \tag{22}$$

$$K_{\text{MD}} = \frac{k_I + k_{\text{off}}}{k_{\text{on}}}, \qquad \alpha = 1 + \frac{\text{N}_{\text{all}} k_I}{k_E}, \tag{23}$$

and k_I, k_E, k_{on}, and k_{off} are the effective transcription initiation, transcription elongation, polymerase-promoter binding, and polymerase promoter dissociation rate constants. In keeping with a previous relation describing the salt dependence of the IVT reaction [7], the rate constant k_{off} was a function of the effective salt concentration [salt],

$$k_{\text{off}} = k_{\text{off,1M}} [\text{salt}]^{n_{\text{salt}}} \tag{24}$$

where $k_{\text{off,1M}}$ is a parameter representing the intrinsic binding strength of the polymerase-promoter complex,

$$[\text{salt}] = \sum_{i=1}^{N_{\text{ion}}} \omega_{\text{ion},i} [\text{ion}_i], \tag{25}$$

$\omega_{\text{ion},i}$ is an ion-specific parameter representing the relative contribution of each ionic complex to the effective salt concentration, and $[\text{ion}_i]$ is the concentration of a given ionic complex i. This equation sums over all N_{ion} complexes in the system, which are enumerated in our speciation model. Nonzero values of ω_{ion} are shown in Table 10. To incorporate the dependence of the IVT reaction on solution conditions, the initiation and elongation rate constants k_I and k_E are dependent on pH, free magnesium concentrations, and NTP concentrations by

$$k_I = \Gamma_{\text{pH}} \Gamma_{\text{Mg}} \Gamma_{\text{NTP}} k_i, \qquad k_E = \Gamma_{\text{pH}} \Gamma_{\text{Mg}} \Gamma_{\text{NTP}} k_e, \tag{26}$$

where k_i and k_e are the intrinsic initiation and elongation rate constants, and

$$\Gamma_{\text{pH}} = \left(1 + \frac{[\text{H}^+]}{K_a} + \frac{K_b}{[\text{H}^+]}\right)^{-1}, \qquad \Gamma_{\text{Mg}} = \frac{[\text{Mg}^{2+}]}{[\text{Mg}^{2+}] + \text{K}_{2,\text{eff}}}, \tag{27}$$

$$\Gamma_{\text{NTP}} = \text{N}_{\text{all}} \left(\sum_{\text{N} \in (\text{A,U,C,G})} \text{N}_{\text{N}} \left(\frac{[\text{MgNTP}^{2-}]}{[\text{MgNTP}^{2-}] + K_1 (1 + [\text{MgPPi}^{2-}]/\text{K}_{\text{i,PPi}})} \right)^{-1} \right)^{-1}. \tag{28}$$

The factors Γ_{Mg} and Γ_{NTP} have an identical structure to previous work [5]. However, it was found that, in order to describe trends in IVT kinetics at high magnesium concentrations, the parameter $K_{2,\mathrm{eff}}$ needed to be higher at higher NTP and Mg concentrations. In keeping with our previous formulation on salt effects, we chose to represent this dependence with a similarly structured power law based on the overall salt concentration (assuming that Mg cannot compete with itself),

$$K_{2,\mathrm{eff}} = K_2([\mathrm{salt}] - \omega_{\mathrm{ion,Mg}}[\mathrm{Mg}^{2+}])^{n_{\mathrm{Mg}}}. \quad (29)$$

The concentrations of individual ionic species, such as the free magnesium concentration $[\mathrm{Mg}^{2+}]$, were calculated by a series of algebraic equations representing the speciation of ions in the IVT solution. The structure and physical assumptions of this model are similar to previous work [5]. In addition to the species in earlier work, this model introduces the complexes of the spermidine species (Sp). In addition, this model considers the binding of magnesium and the phosphate group of RNA ($\mathrm{Pi}^-_{\mathrm{RNA}}$) using an approximate relation based on literature data [8]. The reactions included in this system are collected in Table 9.

Table 9: Parameters of speciation model, where Sp represents spermidine and $\mathrm{Pi}_{\mathrm{RNA}}$ represents the charged phosphate group of RNA.

Speciation Reaction	K (M)
$\mathrm{Tris} + \mathrm{H}^+ \leftrightarrow \mathrm{HTris}^+$	$10^{-8.1}$
$\mathrm{PPi}^{4-} + \mathrm{H}^+ \leftrightarrow \mathrm{HPPi}^{3-}$	$10^{-9.02}$ [9]
$\mathrm{HPPi}^{3-} + \mathrm{H}^+ \leftrightarrow \mathrm{H_2PPi}^{2-}$	$10^{-6.26}$ [9]
$\mathrm{PPi}^{4-} + \mathrm{Na}^+ \leftrightarrow \mathrm{NaPPi}^{3-}$	$10^{-0.21}$ [10]
$\mathrm{NaPPi}^{3-} + \mathrm{Na}^+ \leftrightarrow \mathrm{Na_2PPi}^{2-}$	$10^{0.8}$ [10]
$\mathrm{PPi}^{4-} + \mathrm{Mg}^{2+} \leftrightarrow \mathrm{MgPPi}^{2-}$	$10^{-5.42}$ [9]
$\mathrm{MgPPi}^{2-} + \mathrm{Mg}^{2+} \leftrightarrow \mathrm{Mg_2PPi}$	$10^{-2.33}$ [9]
$\mathrm{HPPi}^{3-} + \mathrm{Mg}^{2+} \leftrightarrow \mathrm{HMgPPi}^-$	$10^{-3.05}$ [9]
$\mathrm{H_2PPi}^{2-} + \mathrm{Mg}^{2+} \leftrightarrow \mathrm{H_2MgPPi}$	$10^{-2.11}$ [11]
$\mathrm{NTP}^{4-} + \mathrm{H}^+ \leftrightarrow \mathrm{HNTP}^{3-}$	$10^{-6.48}$ [12]
$\mathrm{HNTP}^{3-} + \mathrm{Mg}^{2+} \leftrightarrow \mathrm{HMgNTP}^-$	$10^{-2.00}$ [12]
$\mathrm{NTP}^{4-} + \mathrm{Mg}^{2+} \leftrightarrow \mathrm{MgNTP}^{2-}$	$10^{-4.73}$ [9]
$\mathrm{MgNTP}^{2-} + \mathrm{Mg}^{2+} \leftrightarrow \mathrm{Mg_2NTP}$	$10^{-1.69}$ [9]
$\mathrm{NTP}^{4-} + \mathrm{Na}^+ \leftrightarrow \mathrm{NaNTP}^{3-}$	$10^{-1.16}$ [9]
$\mathrm{Sp}^{3+} + \mathrm{NTP}^{4-} \leftrightarrow \mathrm{SpNTP}^-$	$10^{-2.95}$ [13]
$\mathrm{Sp}^{3+} + \mathrm{SpNTP}^- \leftrightarrow \mathrm{Sp_2NTP}^{2+}$	$10^{-2.43}$ [13]
$\mathrm{Pi}^{3-} + \mathrm{H}^+ \leftrightarrow \mathrm{HPi}^{2-}$	$10^{-12.37}$ [12]
$\mathrm{HPi}^{2-} + \mathrm{H}^+ \leftrightarrow \mathrm{H_2Pi}^{1-}$	$10^{-6.92}$ [9]
$\mathrm{HPi}^{2-} + \mathrm{Mg}^{2+} \leftrightarrow \mathrm{HMgPi}$	$10^{-2.20}$ [9]
$\mathrm{HPi}^{2-} + \mathrm{Na}^+ \leftrightarrow \mathrm{HNaPi}^-$	$10^{-0.77}$ [9]
$\mathrm{Pi}^-_{\mathrm{RNA}} + \mathrm{Mg}^{2+} \leftrightarrow \mathrm{MgPi}^+_{\mathrm{RNA}}$	$10^{-2.30+5.07\ln([\mathrm{Na}^+]+[\mathrm{HTris}^+])}$ [8]

Mass balances for the total concentration of each species were used in tandem with these equilibrium relations. In a general case, the total concentration of a species M was

$$[\mathrm{M}]_{\mathrm{tot}} = \sum_{i=1}^{N_{\mathrm{ion}}} p_{\mathrm{M},i}[\mathrm{ion}_i] \quad (30)$$

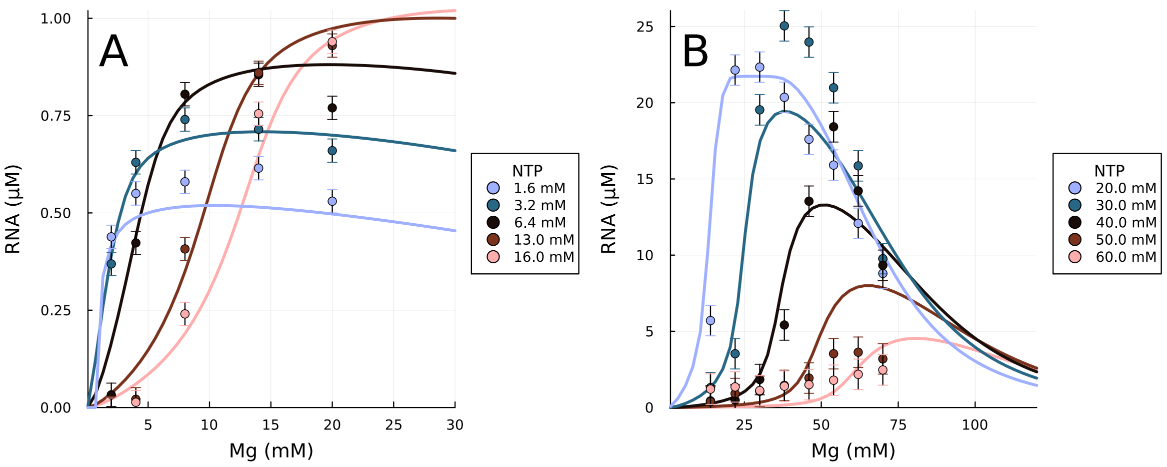

Figure 4: Model fitting results on kinetic IVT data. (A) Data from [14] showing quantity of RNA formed after 5 minutes at varying Mg and NTP input concentrations. (B) Data from [15] showing RNA formed after 1 hour for a higher range of Mg and NTP concentrations.

where $p_{\mathrm{M},i}$ is the number of species M in each complex i. Finally, a charge balance was used to ensure charge neutrality in the ionic system,

$$0 = \sum_{i=1}^{N_{\mathrm{ion}}} c_i[\mathrm{ion}_i] \tag{31}$$

where c_i is the charge of complex i.

5 Parameter Estimation

The model described above was fit to a dataset comprising:

1. The original data set [14] used in previous modeling [5].
2. A set of batch kinetic data from [15] describing the rate of IVT at higher Mg and NTP concentrations.
3. A set of batch kinetic data from [16] describing the effect of pH on IVT kinetics (Figure 2C of the main text).
4. A set of batch kinetic data on the COVID construct used in this work (for estimating sequence-specific initiation and elongation rate constants).
5. The data describing the capping fraction of the RNA product as a function of solution conditions shown in Table 1 of the main text.

The computational methods and statistical assumptions used in parameter estimation were the same as in previous work [5]. Depending on the level of previous knowledge of the model parameters, model sensitivity, and the amount of data present for parameter estimation, model parameters were either fixed, estimated without a prior value, or estimated with a prior value (Table 10). The equilibrium parameters in Table 9 were fixed. To translate between data collected on different DNA sequences, separate initiation elongation and initiation rate constants were estimated corresponding to each dataset.

Parameter	Units	Process	Prior Value	Value after Fitting
$k_{\text{i,akama}}$	h^{-1}	transcription initiation	$10^{3.26\pm0.3}$ [17]	$10^{4.11\pm0.09}$
$k_{\text{i,guo}}$	h^{-1}	transcription initiation	$10^{3.26\pm0.3}$ [17]	$10^{3.29\pm0.05}$
$k_{\text{i,osumi}}$	h^{-1}	transcription initiation	$10^{3.26}$ [17]	–
$k_{\text{i,covid}}$	h^{-1}	transcription initiation	$10^{3.10\pm0.1}$	$10^{3.09\pm0.18}$
$k_{\text{e,akama}}$	h^{-1}	transcription elongation	$10^{5.72\pm0.3}$ [18]	$10^{5.25\pm0.03}$
$k_{\text{e,guo}}$	h^{-1}	transcription elongation	$10^{5.72\pm0.3}$ [18]	$10^{6.21\pm0.59}$
$k_{\text{e,osumi}}$	h^{-1}	transcription elongation	$10^{5.72}$ [18]	–
$k_{\text{e,covid}}$	h^{-1}	transcription elongation	$10^{5.45\pm0.3}$ [18]	$10^{5.46\pm0.09}$
$k_{\text{off,1M}}$	h^{-1}	T7RNAP-DNA binding	–	$10^{2.93\pm0.09}$
$k_{\text{off,1M,covid}}$	h^{-1}	T7RNAP-DNA binding	–	$10^{3.93\pm0.09}$
k_{on}	$h^{-1}nM^{-1}$	T7RNAP-DNA binding	$10^{2.31}$ [19]	–
K_1	M	transcription MgNTP dependence	–	$10^{-3.64\pm0.11}$
K_2	M	transcription Mg dependence	–	$10^{-1.70\pm0.15}$
n_{Mg}	–	transcription Mg dependence	–	$10^{0.26\pm0.04}$
$K_{\text{i,PPi}}$	M	transcription PPi inhibition	–	$10^{-3.90\pm0.12}$
n	–	salt dependence	4.0	–
K_a	M	transcription pH dependence	–	$10^{-7.19\pm0.14}$
K_b	M	transcription pH dependence	–	$10^{-9.28\pm0.10}$
ω_{Na}	–	salt dependence	1 [20]	–
ω_{Mg}	–	salt dependence	4.71 [20]	–
ω_{Cl}	–	salt dependence	0.72 [20]	–
ω_{HTris}	–	salt dependence	1.07 [20]	–
γ	–	capping	–	$10^{-0.71\pm0.59}$
θ	M	capping	–	$10^{-2.53\pm0.79}$

Table 10: Model parameters. Error on the parameter priors represents a 95% confidence interval using standard deviations estimated from literature. Error on parameter posteriors represents the 95% pointwise confidence intervals as approximated by drawing samples from the probability distribution defined by the parameter error covariance matrix.

6 Additional Model Validation

In order to validate the structure and assumptions of the model presented above, model predictions were compared to batch and fed-batch validation data from the literature.

6.1 Kern and Davis (1997) [9]

In addition to data describing the effect of salts on IVT kinetics (Figure 2AB of the main text), the model was validated on data describing the effect of pyrophosphate addition on IVT reaction rates. These data were collected at roughly constant ionic strength and free magnesium concentration, making the data ideal for independent evaluation of the effect of pyrophosphate on the IVT reaction. The general agreement between model prediction and data validates our estimate of $K_{\text{i,PPi}}$ (Figure 5).

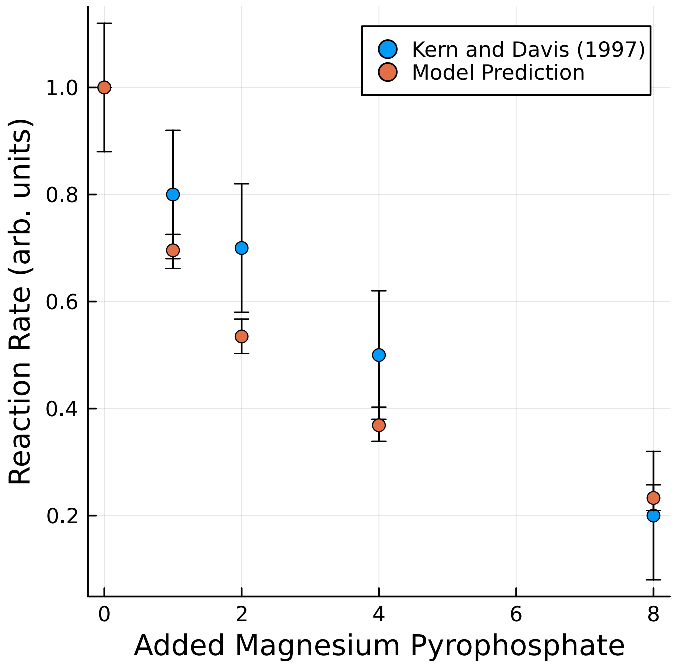

Figure 5: Effect of pyrophosphate addition on IVT reaction rate. Data collected at roughly constant magnesium concentration and ionic strength as detailed in [9].

6.2 Samnuan et al. (2022) [21]

These data were collected as part of a data-driven design of experiments (DoE) to assess the impact of batch IVT conditions on yield of a long (10,000 bp) saRNA sequence. These data were useful for understanding the combined effects of multiple process parameters, including NTP concentrations, magnesium concentrations, and the addition of sodium salts. The transcription rate of this long sequence was found to be roughly linear with respect to RNA polymerase addition, in line with model predictions (Figure 15A). For a specific set of solution conditions, the general nonmonotonic dependence of transcription rates on magnesium input was roughly in line with model predictions (Figure 15B). Finally, general trends in the combined effect of NTP, magnesium, and sodium salt concentrations were captured by our model (Figure 7). Optimal magnesium concentrations were dependent on the concentration of NTP. In addition, the addition of sodium salts generally decreased reaction rates, and the use of chloride rather than acetate salts further decreased rates.

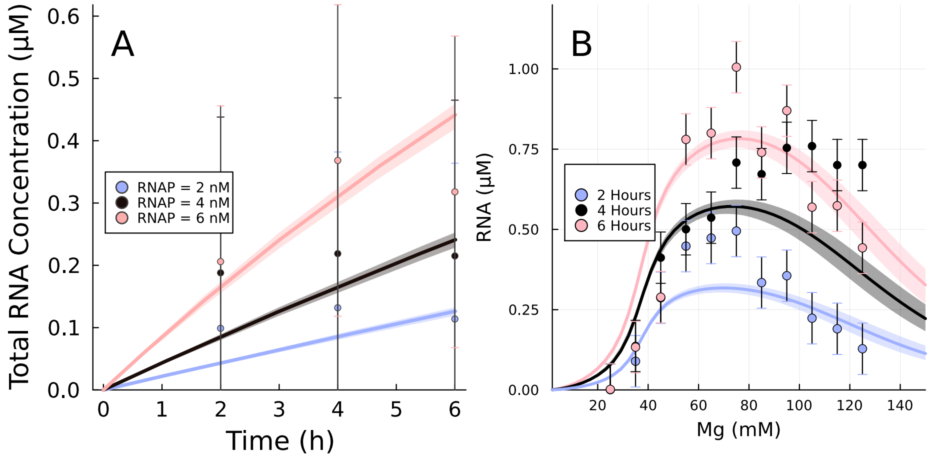

Figure 6: (A) Kinetics of RNA synthesis for different input RNA polymerase concentrations. (B) Transcription yield at 2, 4, and 6 hours as a function of magnesium input. Data from [21].

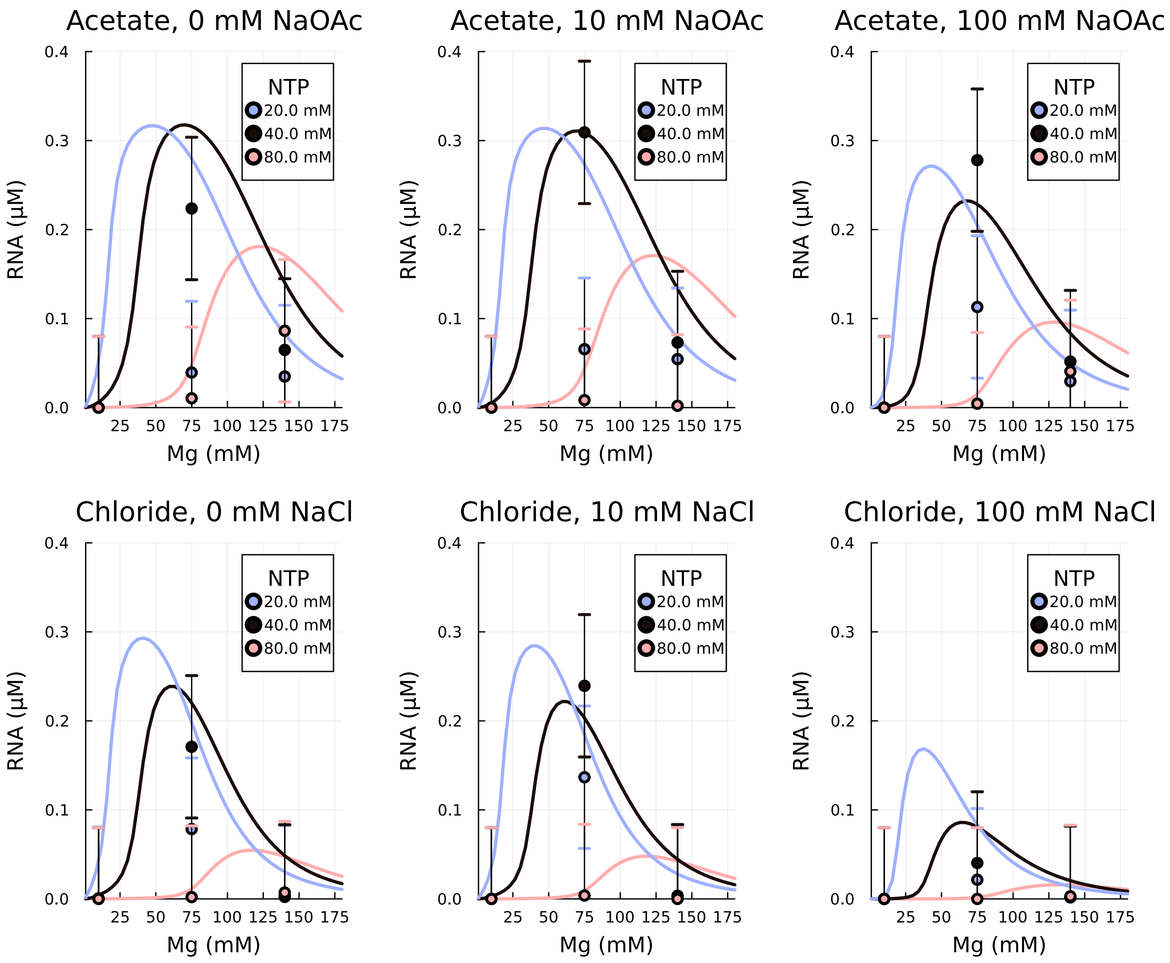

Figure 7: RNA yield after two hours as a function of NTP concentrations, magnesium concentrations, sodium salt input, and choice of acetate or chloride counterion (for both sodium and magnesium salts). Data from [21].

6.3 Pregljc et al. (2023) [2]

These batch and fed-batch data were collected as part of an experimentally driven study on optimization of fed-batch IVT. It was necessary to select a unique k_e ($10^{5.3}$) for the construct used in this work to best represent these data. Our model generally predicts trends in the effect of magnesium on IVT kinetics, but failed to predict a halting in the reaction at very high (50 mM) magnesium concentrations (Figure 8AB). The model generally predicted the phenomena that the increase of NTP concentrations at a constant magnesium concentration greatly decreased reaction rates (Figure 8C). In line with these batch results, the model successfully predicts the failure of a fed-batch feeding strategy that lacked magnesium relative to a strategy that included magnesium (Figure 9). The model generally predicts the RNA and NTP concentration trajectories of a number of different fed-batch strategies (Figure 10). In particular, the model successfully predicts the observed decrease in reaction rate at later stages of the reaction.

Finally, Pregeljc et al. [2] tested a number of strategies for the incorporation of co-transcriptional capping to fed-batch IVT using a binucleoside cap (ARCA) (Figure 11). In addition to concentration trajectories of RNA and NTPs, we used our model to predict cap fractions based on equation 3 using an estimated value γ_{bi} of one. The model prediction that the decrease of solution GTP setpoints during fed-batch operation would increase the final

cap fraction was roughly in line with experimental observations.

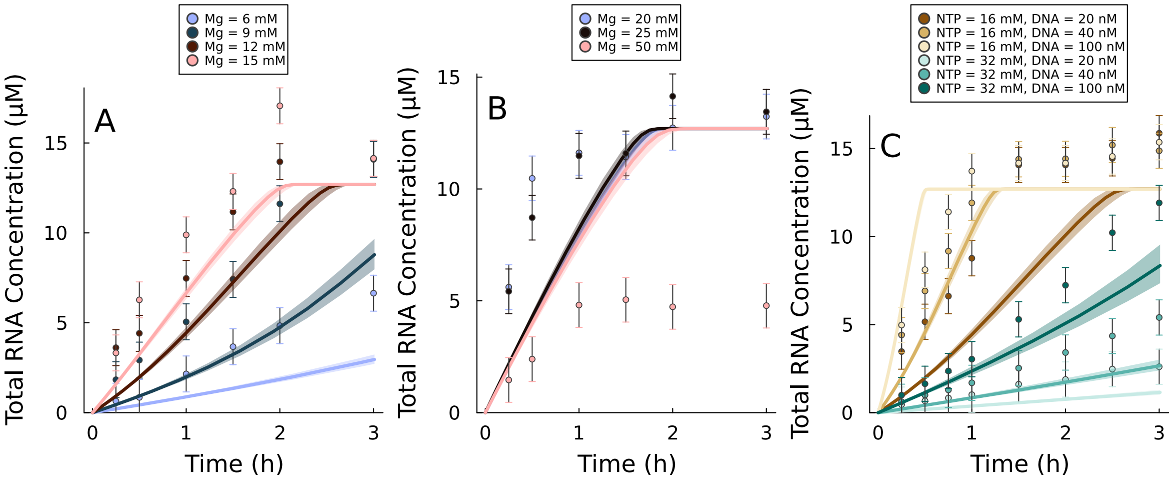

Figure 8: (A,B) Transcription kinetics as a function of magnesium input with other solution conditions held constant. (C) Transcription kinetics as a function of DNA and NTP input concentrations with other solution conditions held constant. Data from [2].

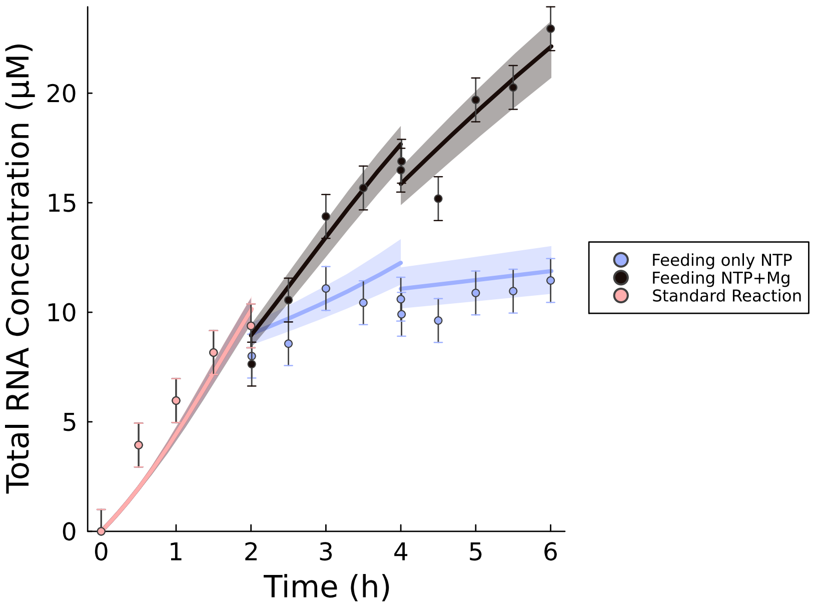

Figure 9: RNA concentration in fed-batch reactions over time. A strategy of feeding NTP is compared with joint feeding of NTP and magnesium. Initial conditions are the same between the two experiments. Shaded areas represent 95% model uncertainty intervals based on Monte Carlo sampling of covariance matrix. Data from [2].

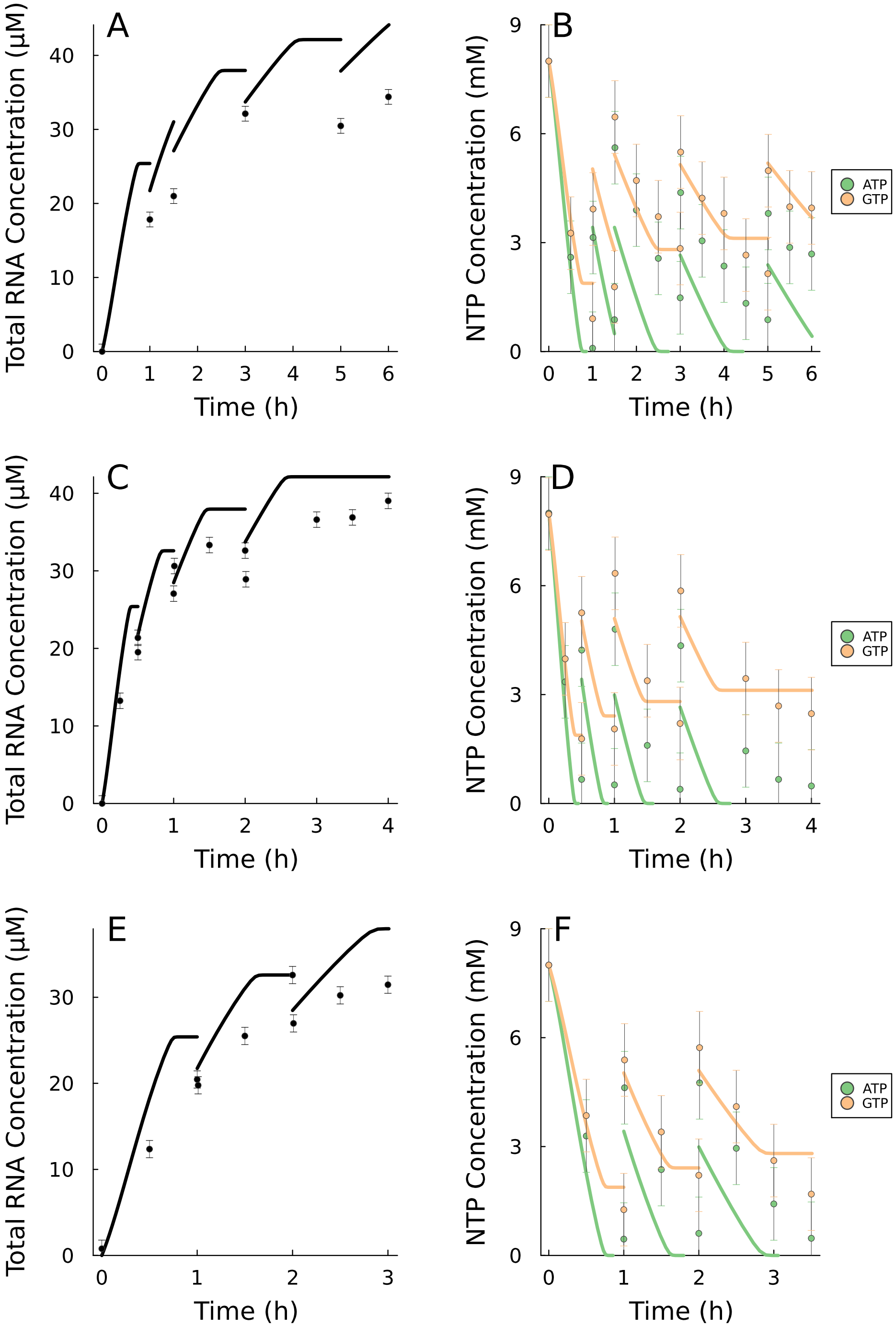

Figure 10: Fed-batch strategies from [2] that vary in reaction timescale, feeding quantity, and feeding composition. (The three strategies shown come from Figures 4AB, SI(CD), and 5EF of the original work). RNA (ACE) and NTP (BDF) concentration trajectories are shown for each strategy.

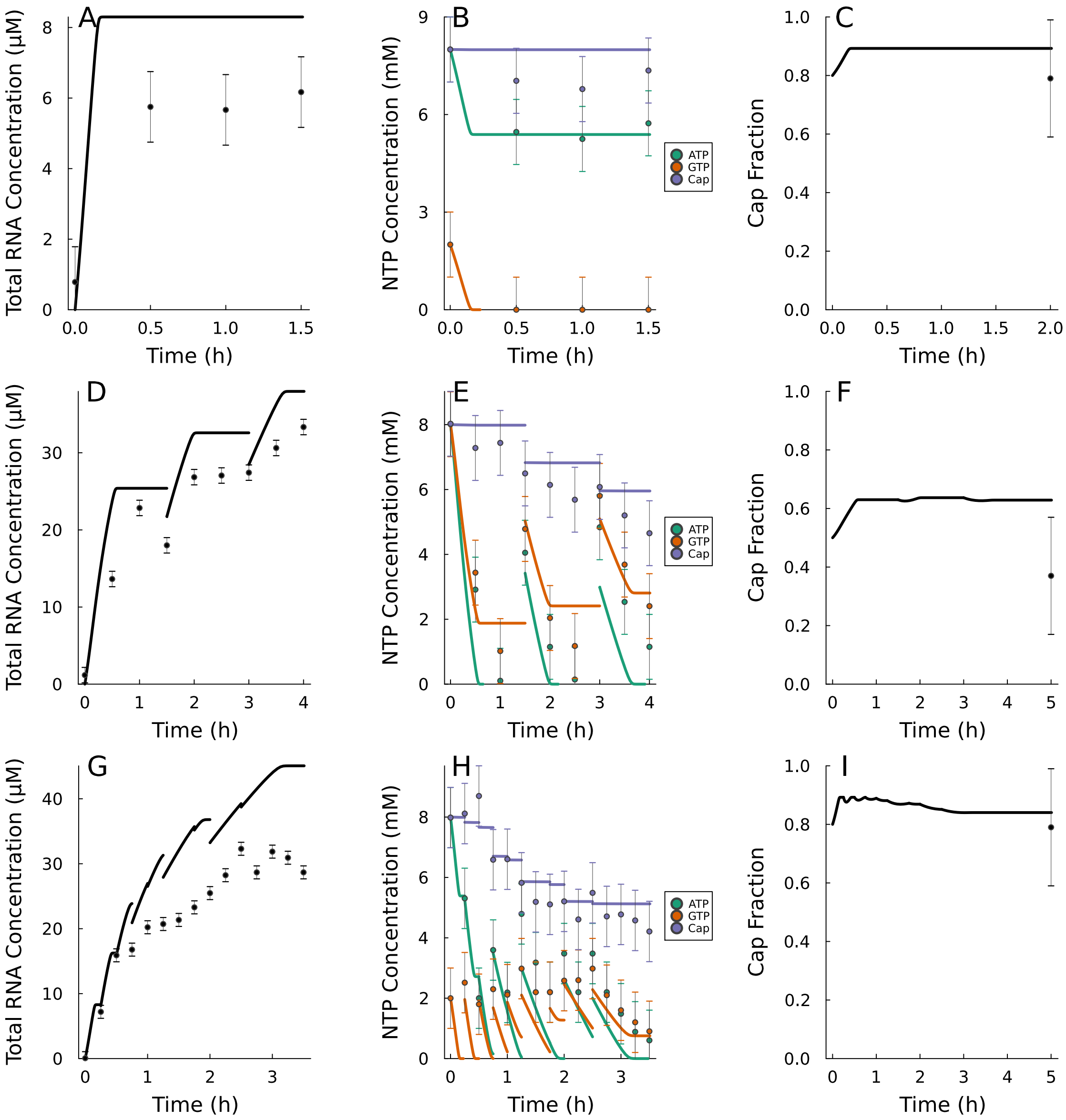

Figure 11: Batch and fed-batch co-transcriptional capping data from [2]. RNA and NTP concentration trajectories are shown for each experiment in addition to the cap fraction. The three experiments shown represent a batch reaction (ACB), a fed-batch reaction targeting a GTP concentration of approximately 2–4 mM (DEF) and a fed-batch reaction targeting a GTP concentration of approximately 0–2 mM (GHI).

6.4 Kern and Davis (1999) [6]

These data were collected on the fed-batch synthesis of oligomeric RNA using a unique form of feedback control that sought to control both NTP concentrations and pH based on pH measurements. These data were used to validate our approach for modeling the decline in reaction rate during fed-batch operation. Because the pH of these reactions were controlled, the observed decline in reaction rate cannot be attributed to pH effects. The model captures the general trends in the decline of reaction rate in both the synthesis of 12-mer (Figure 12) and 38-mer (Figure 13) RNA.

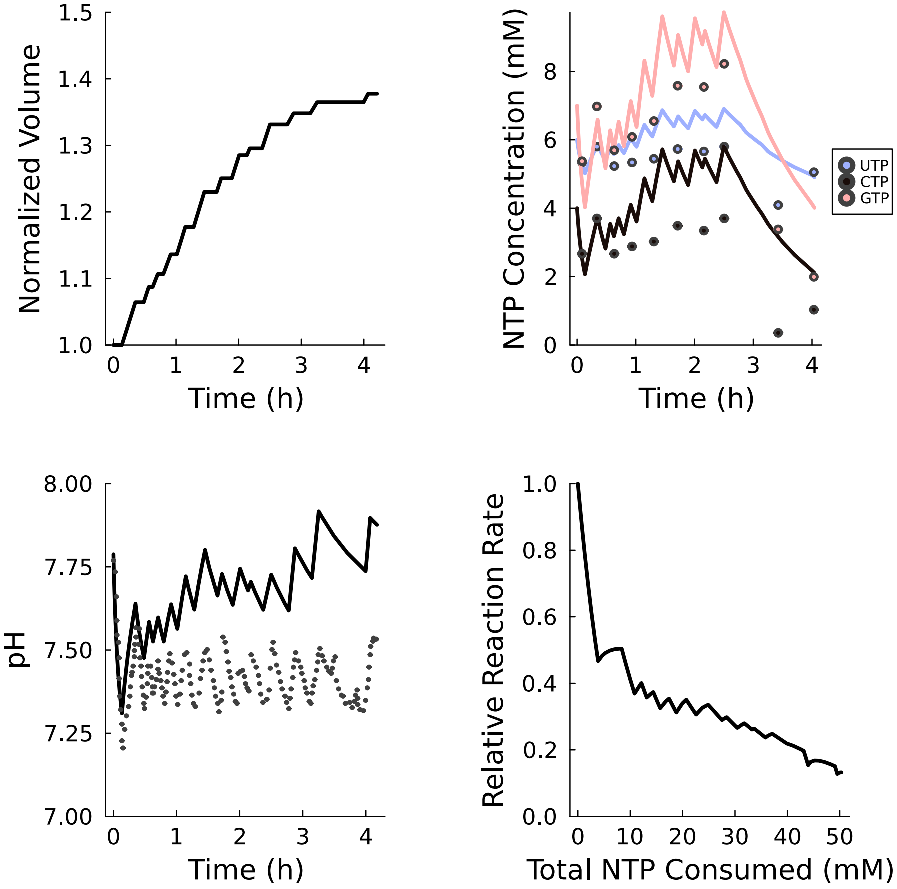

Figure 12: Fed-batch synthesis of 12-mer oligomeric RNA using pH control. Feeding profile, NTP concentrations, pH, and model predictions of reaction rate as a function of reaction progression are shown. Data from [6].

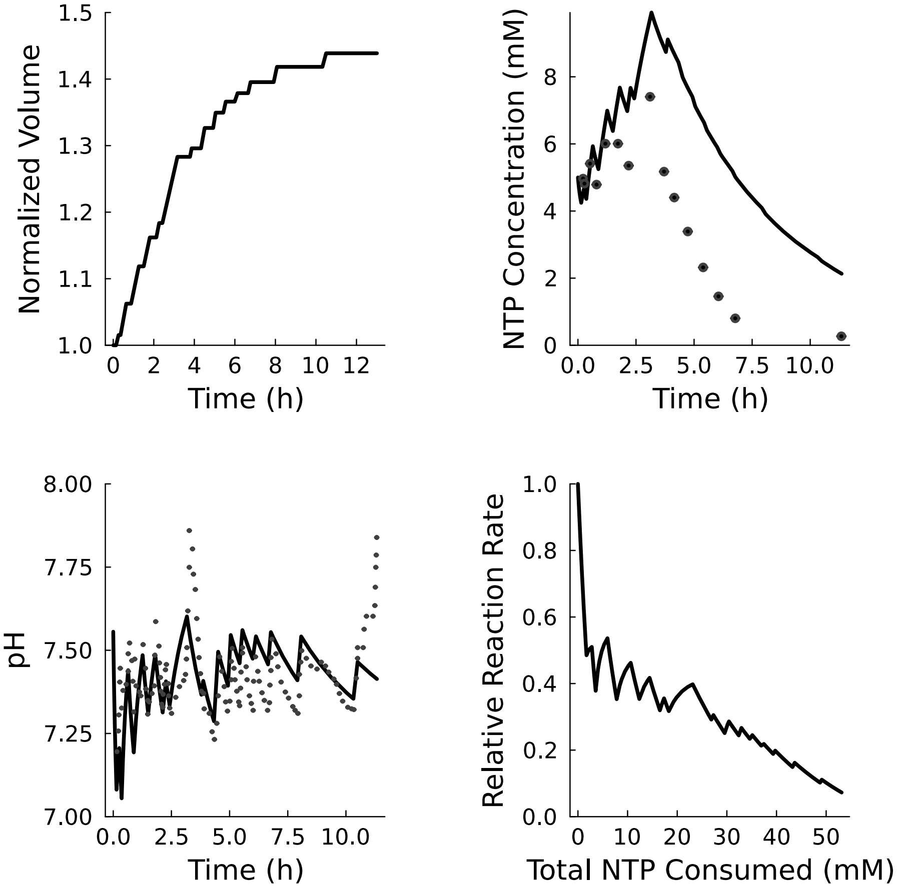

Figure 13: Fed-batch synthesis of 38-mer oligomeric RNA using pH control. Feeding profile, NTP concentrations, pH, and model predictions of reaction rate as a function of reaction progression are shown. Data from [6].

6.5 Skok et al. (2022) [22]

These data comprise two fed-batch reactions: one with discrete addition of feed, and the other using a complex continuous feeding profile. We estimated initiation and elongation rate constants based on the discrete-fed batch reaction (Figure 14), and validated this choice on the continuously fed fed-batch reaction (Figure 2 of the main text).

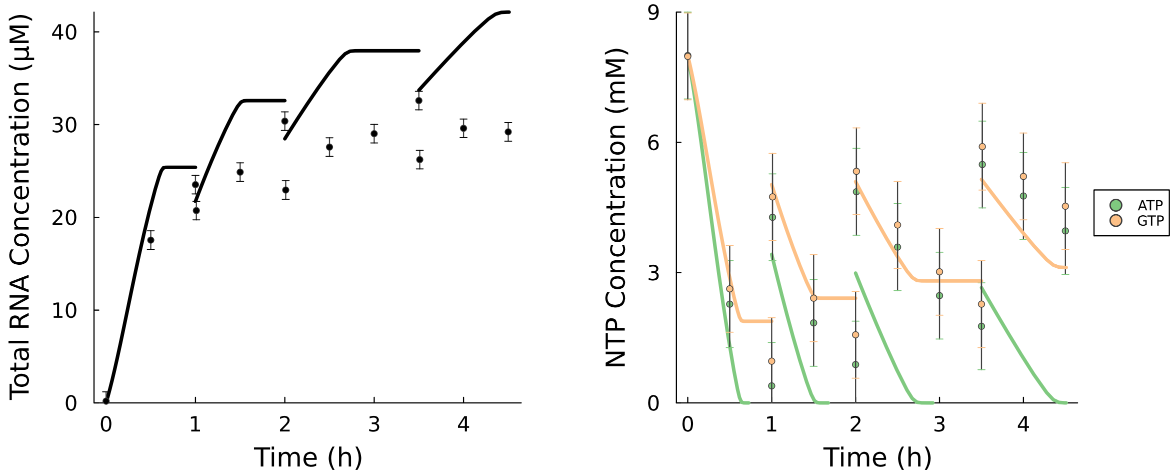

Figure 14: Discrete fed-batch reaction from [22] used for estimating kinetic parameters for use modeling continuously fed fed-batch reaction (Figure 2D of the main text).

6.6 Elich et al. [4] (2022)

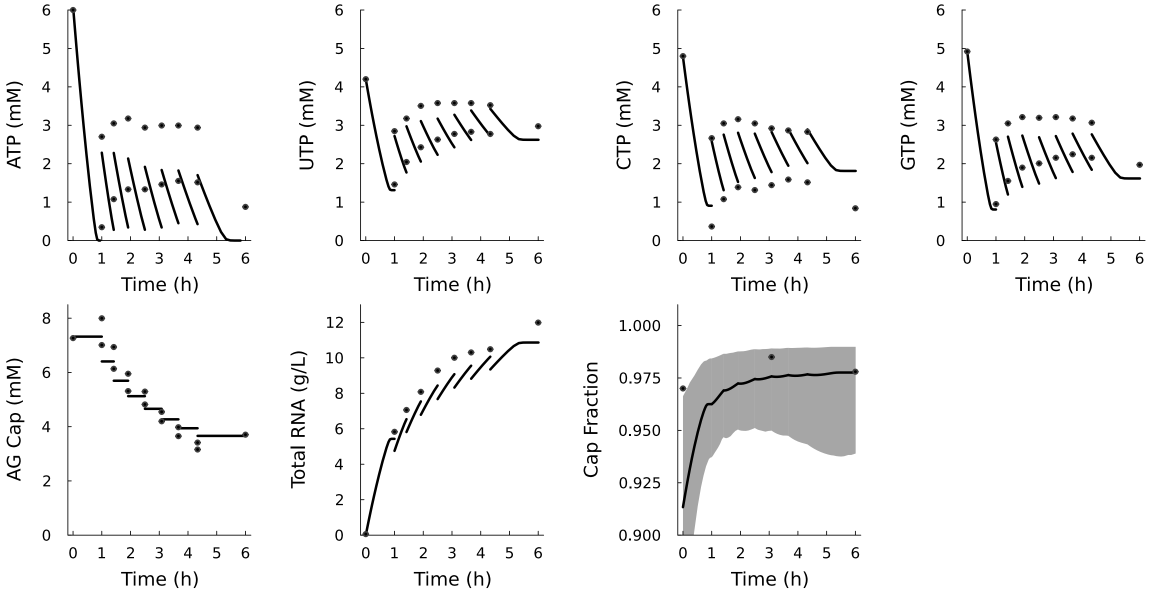

Figure 15: Data originally published as Figures 11–16 describing the synthesis of "RNA 1" in Elich et al. (2022). Model applied to published data showing dynamics of NTP concentrations, AG cap concentrations, RNA concentrations, and the cap fraction. The model predictions shown are using an adjusted initiation rate constant of 984 h^{-1} to best reflect the initial rate of these data. Model predictions for cap fraction use the same parameters as estimated in the main text.

6.7 Boman et al. (2024)

Boman et al. [23] performed a data-driven analysis and optimization of IVT. One key portion of this dataset was a comparison of the effect of changing the magnesium counterion from chloride to acetate. Figure 16 shows the predictions of the model (using the kinetic parameters estimated for the COVID construct used in this work) on kinetic data collected by Boman et al. comparing these two counterion strategies. For all conditions shown except for the case of

high NTP concentrations and low Mg concentrations, the model generally predicts both the rates of reaction and the relative different in reaction rate between the acetate and chloride cases. In the case of other kinetic data from Boman et al. collected at very high concentrations of NTPs (>50 mM), the model dramatically under-predicted reaction rates by a factor of 10^2. This shows that with regard to the relationship of magnesium and NTP on reaction rates (the most empirical portion of this model), the model is only valid in the range of the fitting data.

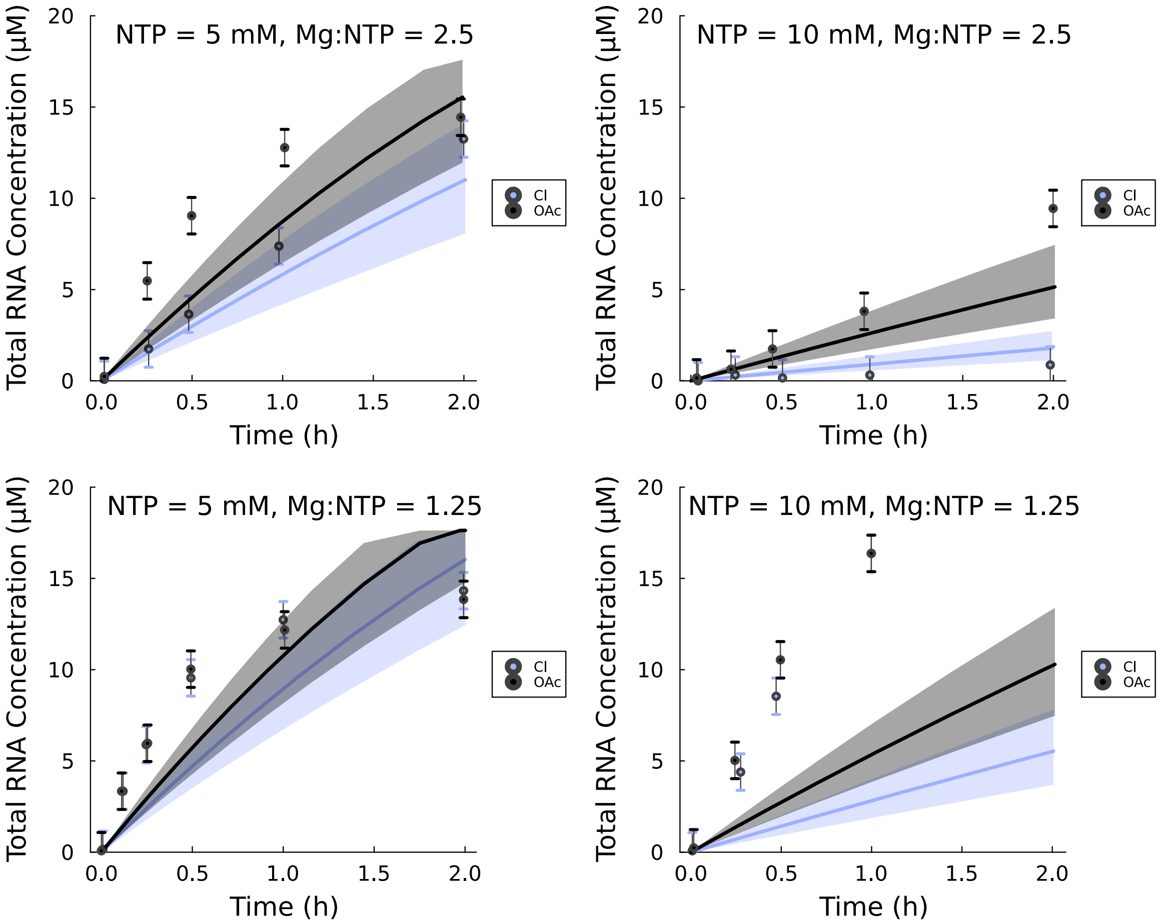

Figure 16: Data from Boman et al. [23] comparing the effect of cloride and acetate magnesium countertions on IVT reaction kinetics. The four reaction conditions shown cover a range of magnesium and NTP concentrations and correpond to those in Figure 7a of Boman et al.

7 Dynamics of Magnesium Phosphate Precipitation

Figure 4 of the main text compares the final state of low-pH and high-pH strategies for control of fed-batch IVT reactions. These data show the dynamics of these same measurements for the entire period of process operation.

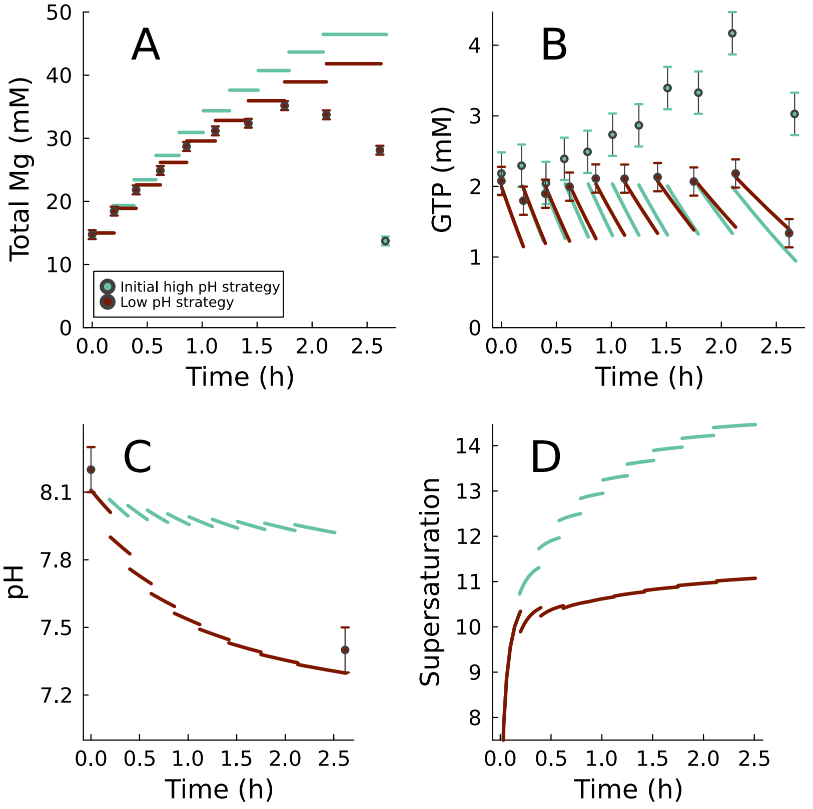

Figure 17: Comparing magnesium phosphate precipitation during high-pH (targeting pH 8.0) and low-pH (7.3) strategies for fed-batch IVT. (A) Total solution magnesium concentration over time. Lines represent model prediction assuming no precipitation. (B) GTP concentration (C) pH, and (D) thermodynamic supersaturation of magnesium phosphate over time.

8 Characterization of Magnesium Phosphate Precipitate

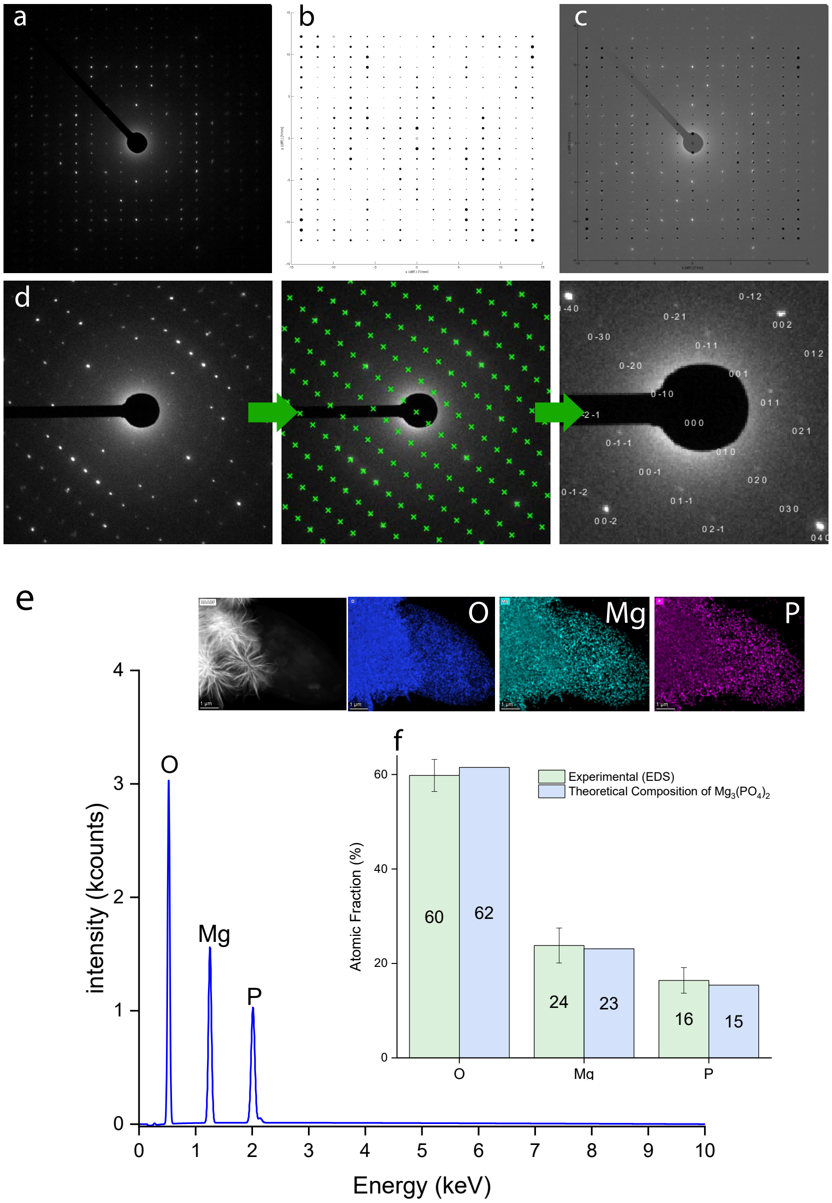

Figure 18: (a) Experimental electron diffraction pattern. (b) Simulated diffraction pattern along the [100] zone axis of anhydrous magnesium phosphate [24], generated using the CrysTBox cellViewer tool [25]. (c) Overlay of experimental and simulated patterns for visual comparison. (d) Indexed diffraction pattern produced using the CrysTBox diffractGUI tool. (e) STEM-EDS elemental map with corresponding spectra. (f) Quantitative comparison between experimentally measured and theoretically calculated compositions; values are presented in atomic percent (mole-based).

9 Counterfactual Simulation of Heuristic Process Assuming a Lower Salt Sensitivity

In order to model the experimental data shown in this work, the parameter $k_{\text{off,1M}}$ was decreased from the canonical value shown in Table 10 by a factor of 10. This adjustment was

performed based off of previous data describing the effect of sodium chloride on the reaction kinetics of this construct [7]. Had this salt sensitivity not been present, our model predictions indicate that the heuristic process would have maintained NTP concentrations to a narrower window and produced greater quantities of RNA.

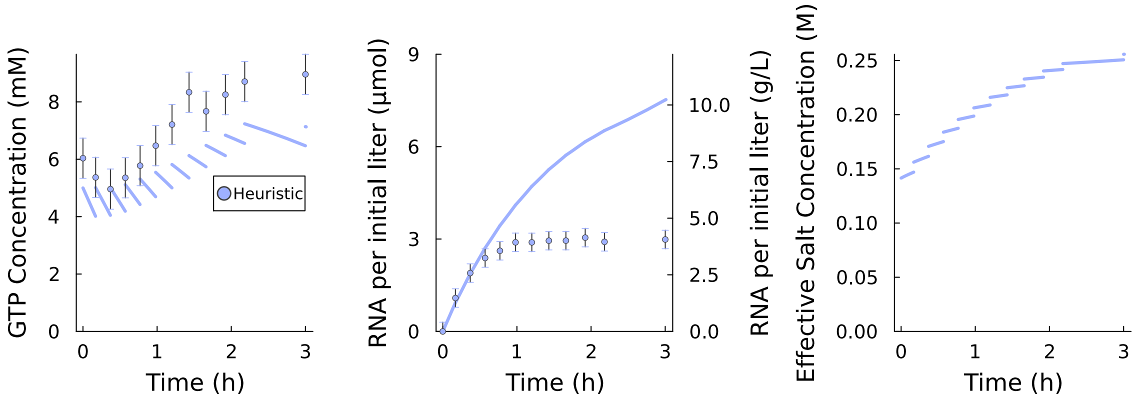

Figure 19: Model prediction of heuristic process (Figure 3 of the main text) assuming a value of $k_{\mathrm{off,1M}}$ equal to that shown in Table 10.

10 Comparison with Previous Experimental Results

We compare our results with representative best results from previous work. For Elich et al. (2022), we compare our results to the synthesis of "RNA 1". For Pregeljc et al. (2023), we compare with the reaction shown in Figure 6b of their work.

Table 11: Comparison with fed-batch results from previous work

	This work	Elich et al. (2022) [4]	Pregeljc et al. (2023) [2]	Skok et al. (2022) [22]
On-line measurement required	No	No	Yes	Yes
Reaction time (h)	2.5	6	2.5	7
Range of ATP concentrations (mM)	1.25–2.5	0.25–6	2–8	0.37–8
RNA produced per initial liter (g/L)	10	24	13	21
RNA produced per inial liter after 2.5 hours (g/L)	10	18	13	13.5
RNA polymerase per initial liter (nM)	100	800	unknown	160
DNA per initial liter (g/L)	0.05	0.05	unknown	0.05
RNA after 2.5 hours per RNA polymerase (g/L nM)	0.10	0.023	unknown	0.084
RNA produced (g/L) after 2.5 hours per g/L of DNA	80	80	unknown	60
Cap Type	Trinucleoside AG	Trinucleoside AG	Binucleoside G	-
Cap per initial liter (mM)	2	7.2	8	-
Final cap fraction	0.88±0.05	0.975	0.67	-

References

[1] E. N. Welbourne, K. A. Loveday, A. Nair, *et al.*, "Anion exchange HPLC monitoring of mRNA in vitro transcription reactions to support mRNA manufacturing process development," English, *Frontiers in Molecular Biosciences*, vol. 11, 2024.

[2] D. Pregeljc, J. Skok, T. Vodopivec, *et al.*, "Increasing yield of in vitro transcription reaction with at-line high pressure liquid chromatography monitoring," en, *Biotechnology and Bioengineering*, vol. 120, no. 3, pp. 737–747, 2023.

[3] D. C. Liu and J. Nocedal, "On the limited memory BFGS method for large scale optimization," en, *Mathematical Programming*, vol. 45, no. 1, pp. 503–528, 1989.

[4] J. Elich, A. E. Rabideau, M. Shamashkin, R. Philpot, B. Fritz, and P. Wojciechowski, "Fed-batch in vitro transcription process," US20220145381A1, 2022.

[5] N. M. Stover, K. Ganko, and R. D. Braatz, "Mechanistic modeling of in vitro transcription incorporating effects of magnesium pyrophosphate crystallization," en, *Biotechnology and Bioengineering*, vol. 121, no. 9, pp. 2636–2647, 2024.

[6] J. A. Kern and R. H. Davis, "Application of a Fed-Batch System To Produce RNA by In Vitro Transcription," en, *Biotechnology Progress*, vol. 15, no. 2, pp. 174–184, 1999.

[7] N. M. Stover, M. D. Bock, J. Chen, *et al.*, *Emergent kinetics of in vitro transcription from interactions of T7 RNA polymerase and DNA (preprint)*, 2025.

[8] H. Krakauer, "The binding of Mg++ ions to polyadenylate, polyuridylate, and their complexes," en, *Biopolymers*, vol. 10, no. 12, pp. 2459–2490, 1971.

[9] J. A. Kern and R. H. Davis, "Application of solution equilibrium analysis to in vitro RNA transcription," eng, *Biotechnology Progress*, vol. 13, no. 6, pp. 747–756, 1997.

[10] R. M. Smith and A. E. Martell, *Critical Stability Constants*, en. Boston, MA: Springer US, 1976, ISBN: 978-1-4757-5508-4 978-1-4757-5506-0.

[11] J. S. Young, W. F. Ramirez, and R. H. Davis, "Modeling and optimization of a batch process for in vitro RNA production," en, *Biotechnology and Bioengineering*, vol. 56, no. 2, pp. 210–220, 1997.

[12] R. A. Alberty and R. N. Goldberg, "Standard thermodynamic formation properties for the adenosine 5'-triphosphate series," *Biochemistry*, vol. 31, no. 43, pp. 10 610–10 615, 1992.

[13] T. Günther, J. Vormann, P. Konstanczak, and A. Schäfer, "Interactions of polyamines in the measurement of free magnesium concentration by mag-fura-2 and 31P-NMR," *Biochimica et Biophysica Acta (BBA) - Biomembranes*, vol. 1192, no. 2, pp. 281–285, 1994.

[14] S. Akama, M. Yamamura, and T. Kigawa, "A Multiphysics Model of In Vitro Transcription Coupling Enzymatic Reaction and Precipitation Formation," en, *Biophysical Journal*, vol. 102, no. 2, pp. 221–230, 2012.

[15] L. Guo, Z. Liu, S. Song, W. Yao, M. Yang, and G. Chen, "Maximizing the mRNA productivity for in vitro transcription by optimization of fed-batch strategy," *Biochemical Engineering Journal*, vol. 210, p. 109 412, 2024.

[16] P. A. Osumi-Davis, N. Sreerama, D. B. Volkin, C. R. Middaugh, R. W. Woody, and A.-Y. M. Woody, "Bacteriophage T7 RNA Polymerase and Its Active-site Mutants: Kinetic, Spectroscopic and Calorimetric Characterization," *Journal of Molecular Biology*, vol. 237, no. 1, pp. 5–19, 1994.

[17] M. Maslak and C. T. Martin, "Effects of solution conditions on the steady-state kinetics of initiation of transcription by T7 RNA polymerase," eng, *Biochemistry*, vol. 33, no. 22, pp. 6918–6924, 1994.

[18] G.-Q. Tang, V. S. Anand, and S. S. Patel, "Fluorescence-Based Assay to Measure the Real-time Kinetics of Nucleotide Incorporation during Transcription Elongation," en, *Journal of Molecular Biology*, vol. 405, no. 3, pp. 666–678, 2011.

[19] H. R. Koh, R. Roy, M. Sorokina, *et al.*, "Correlating Transcription Initiation and Conformational Changes by a Single-Subunit RNA Polymerase with Near Base-Pair Resolution," English, *Molecular Cell*, vol. 70, no. 4, 695–706.e5, 2018.

[20] T. Łoziński and K. L. Wierzchowski, "Evaluation of mixed-salt effects on thermodynamic and kinetic parameters of RNA polymerase-promoter DNA complexes in terms of equivalent salt concentrations. General applicability to DNA complexes," eng, *Acta biochimica Polonica*, vol. 56, no. 4, pp. 695–702, 2009.

[21] K. Samnuan, A. K. Blakney, P. F. McKay, and R. J. Shattock, "Design-of-experiments *in vitro* transcription yield optimization of self-amplifying RNA," en, *F1000Research*, 2022.

[22] J. Skok, P. Megušar, T. Vodopivec, *et al.*, "Gram-Scale mRNA Production Using a 250-mL Single-Use Bioreactor," en, *Chemie Ingenieur Technik*, vol. 94, no. 12, pp. 1928–1935, 2022.

[23] J. Boman, T. Marušič, T. V. Seravalli, *et al.*, "Quality by design approach to improve quality and decrease cost of in vitro transcription of mRNA using design of experiments," en, *Biotechnology and Bioengineering*, vol. 121, no. 11, pp. 3415–3427, 2024.

[24] A. Nord and P. Kierkegaard, "The crystal structure of Mg3(PO4)2," *Acta Chemica Scandinavica*, vol. 22, no. 1466, 1968.

[25] M. Klinger and A. Jäger, "Crystallographic Tool Box (CrysTBox): Automated tools for transmission electron microscopists and crystallographers," en, *Journal of Applied Crystallography*, vol. 48, no. 6, pp. 2012–2018, 2015.
